# Sex-specific circulating unconventional neutrophils determine immunological outcome of auto-inflammatory Behçet’s uveitis

**DOI:** 10.1038/s41421-024-00671-2

**Published:** 2024-05-04

**Authors:** Qingfeng Wang, Junfeng Ma, Yuxing Gong, Lifu Zhu, Huanyu Tang, Xingsheng Ye, Guannan Su, Fanfan Huang, Shiyao Tan, Xianbo Zuo, Yuan Gao, Peizeng Yang

**Affiliations:** 1 The First Affiliated Hospital of Chongqing Medical University, Chongqing, China; 2 Southwest Hospital/Southwest Eye Hospital, Third Military Medical University, Chongqing, China; 3 Key Lab of Visual Damage and Regeneration & Restoration of Chongqing, Chongqing, China; 4 Translational Medicine Research Center, Shanxi Medical University, Taiyuan, Shanxi China; 5 China-Japan Friendship Hospital, Beijing, China, and No. 1 Hospital, Anhui Medical University, Anhui, China

**Keywords:** Autoimmunity, Innate immunity

## Abstract

Neutrophils are the most abundant immune cells that first respond to insults in circulation. Although associative evidence suggests that differences in neutrophils may be linked to the sex-specific vulnerability of inflammatory diseases, mechanistic links remain elusive. Here, we identified extensive sex-specific heterogeneity in neutrophil composition under normal and auto-inflammatory conditions at single-cell resolution. Using a combination of single-cell RNA sequencing analysis, neutrophil-specific genetic knockouts and transfer experiments, we discovered dysregulation of two unconventional (interferon-α responsive and T cell regulatory) neutrophil subsets leading to male-biased incidence, severity and poor prognosis of auto-inflammatory Behçet’s uveitis. Genome-wide association study (GWAS) and exosome study revealed that male-specific negative effects of both genetic factors and circulating exosomes on unconventional neutrophil subsets contributed to male-specific vulnerability to disease. Collectively, our findings identify sex-specifically distinct neutrophil subsets and highlight unconventional neutrophil subsets as sex-specific therapeutic targets to limit inflammatory diseases.

## Introduction

Disease affects females and males differently, and sex-specific health differences are of particular interest^1^. Differences in immune responses between females and males are well-recognized but ambiguous. Females have more effective immune responses fighting against immunization and infection, but are more likely to develop an autoimmune disease^2,3^, with 80 percent of autoimmune disease cases diagnosed in females^4^. Males are more prone to neurodegenerative diseases and cardiovascular diseases such as Parkinson’s disease and myocardial infarction^5,6^. Some of these cases may come down to genetic differences that are the results of differential X-chromosome inactivation, sex hormone level, gender, behavior, or life experiences^7^. Identifying mechanisms underlying sex-related differences is essential for developing approaches to effectively modulate immune responses.

The cellular immune response is an essential component of immune defense. Neutrophils, as first effector cells of the innate immune system, are involved in a diverse array of immunological and inflammatory processes^8,9^. Under homeostatic conditions, neutrophils patrol the blood and tissues for the detection of stimuli, where they contribute to various physiological functions, including angiogenesis, coagulation and tissue repair^10–12^. Different from autoimmune diseases, auto-inflammatory diseases are a group of inflammatory diseases characterized by recurrent and systemic inflammation caused by dysregulation of the innate immune system instead of perturbations in adaptive immune system^13^. Behçet’s disease is a heterogeneous auto-inflammatory vasculitis mainly involving neutrophils, with elevated prevalence in men and individuals of non-Swedish ancestry along the silk route^14^. Uveitis is an intraocular inflammation which is one of the most severe and frequent manifestations of Behçet’s disease^15^. Although neutrophils have recently been reported as a critical factor in the pathogenesis of several systemic auto-inflammatory diseases, including but not limited to Behçet’s uveitis (BU)^16^, the study of their sex-specific differences has been reported rarely^17^. Bulk transcriptomic profiling of circulating neutrophils from BU patients has implicated aberrant type I interferon signaling, dysregulated neutrophil activation, and failure of apoptotic neutrophil clearance as hallmarks of disease^18,19^. Single-cell gene expression analysis has indicated that neutrophils are significantly more versatile and heterogeneous than previously considered^20^. Despite this progress, a comprehensive census of circulating neutrophils underlying sex-specific single cell variabilities in homeostatic/auto-inflammatory conditions remains incomplete, and annotating the neutrophil subset types and cell contexts mediating genetic associations, as well as the effects of circulating environmental factors remain challenging.

In this study, we applied single-cell RNA sequencing (scRNA-seq) to circulating neutrophils from healthy individuals and auto-inflammatory BU patients to describe the sex-specific heterogeneity in neutrophil composition that was associated with immunological outcome of BU. More specifically, our work identified unconventional neutrophil subsets as a major determinant of male-biased vulnerability to BU, and revealed that IFN-α2a could directly promote unconventional neutrophil subsets to a more immune-regulatory phenotype. We also determined the negative effects of both genetic factors and circulating exosomes on unconventional neutrophil subsets, which also contributed to male-biased vulnerability to disease. Our work shed new light on the cellular processes of neutrophils contributing to sex-specifically immunological outcome of BU, which might guide the development of rationally sex-specific immunotherapies targeting neutrophils to benefit patients with auto-inflammatory diseases.

## Results

### ScRNA-seq analysis identifies sex-specific neutrophil subsets

**Fig. 1:**
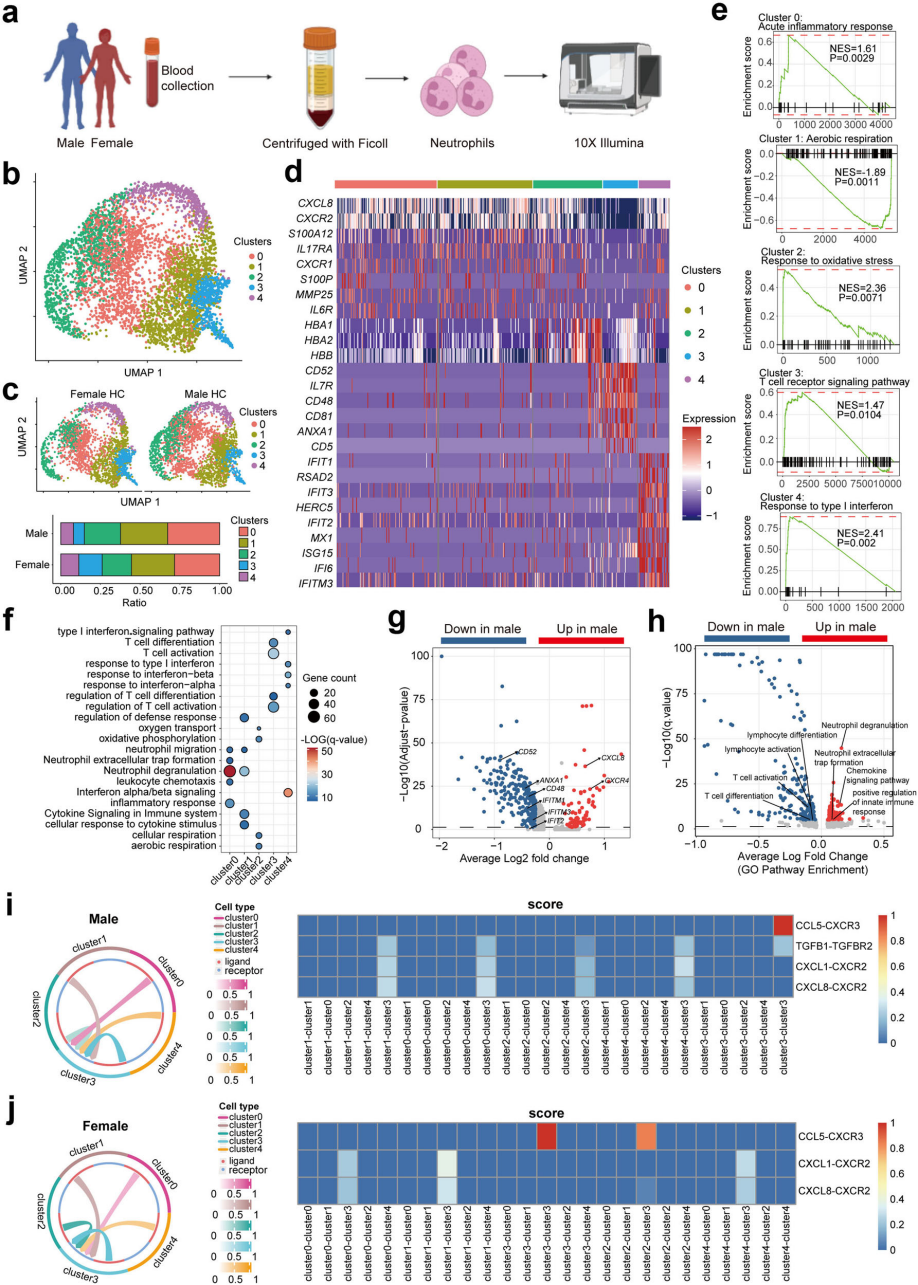
scRNA-seq analysis identifies distinct neutrophil subsets with sex-specific heterogeneity. **a** Schematic representation of the experimental design for scRNA-seq analysis of neutrophils from female HCs (*n* = 8) and male HCs (*n* = 8). **b** UMAP visualization of 5341 neutrophils from female and male HCs. **c** Upper panel: UMAP visualization of human neutrophils; lower panel: bar plot of the proportions of different neutrophil subsets shown in female HCs and male HCs. **d** Heatmap showing the expression of selected cluster marker genes. **e** GSEA identifying the properties of 5 neutrophil subsets. **f** Dotplot heatmap showing GO biological process terms enriched in cluster marker genes. **g** Volcano plot showing the DEGs of neutrophils between female and male HCs. **h** GO analysis of the DEGs of neutrophils. **i**, **j** Left panel: Circos plots of inter-cellular communication among different neutrophil subsets from female and male HCs; right panel: heatmap showing the scores of different L-R pairs between neutrophil subsets. *n* refers to the number of each group.

To fully capture the sex-specific molecular repertoire of neutrophils at single-cell resolution, we first conducted scRNA-seq on circulating neutrophils from healthy female and male donors using the 10x Genomics Chromium platform. In detail, we collected neutrophils from gradient centrifugation of peripheral blood and also used fluorescence-activated cell sorting (FACS) to enrich for CD15^+^CD11b^+^ neutrophils. In order to capture more neutrophils with higher RNA content, we sorted neutrophils within 3 h from the fresh peripheral blood and pooled cells from 2–4 donors (females and males, respectively) with equal cell numbers before processing them for scRNA-seq (Fig. 1a). After data pre-processing and quality control, a total of 5341 neutrophils from 8 females and 8 males were partitioned into 5 clusters (cluster 0–cluster 4) that expressed neutrophil-specific genes (*S100A8* and *S100A9*) using two-dimensional uniform manifold approximation and projection (UMAP) (Fig. 1b). The proportions of neutrophil subsets varied between females and males. Relatively higher abundances of cluster 0, 1 and 2 were observed in males, whereas the proportions of cluster 3 and 4 were decreased in males as compared with females (Fig. 1c). We then analyzed the gene signatures of these neutrophil subsets respectively, and identified differential expressed genes (DEGs) across different subsets (Fig. 1d; Supplementary Table S3). The genes of cluster 0 and cluster 1 were associated with inflammatory responses (*S100P*, *MMP25*, *IL6*, and *S100A12*), and regulators of chemotaxis function (*CXCR2*, *CXCR1*, and *CXCL8*). Highly expressed genes of cluster 2 were associated with cellular respiration (*HBA1*, *HBA2*, and *HBB*). In contrast to clusters 0, 1 and 2, genes of cluster 3 were predominantly enriched in the pathway of regulation of T cell differentiation (*CD52*, *ANXA1*, *IL7R*, and *CD81*). And cluster 4 was a unique subset of neutrophils that expressed higher levels of type I interferon-stimulated genes (ISGs). Our data were consistent with previous investigation^21^ that a subset of neutrophils with an IFN-α-responsiveness state was also identified by single-cell gene expression. More importantly, neutrophils from female donors had higher expression of a key set of ISGs responsible for regulating responsiveness to stimulation and subsequent effector function, including COVID-19, autoimmunity and cancer^3,22,23^. Simultaneously, decreased expression of genes that were associated with inflammatory responses and neutrophil chemotaxis function was observed in both cluster 3 and cluster 4 (Fig. 1d) as compared with cluster 0, 1 and 2, indicating that these two subsets were less “inflammatory”phenotype.

In order to further explore the intrinsic function of these subsets of neutrophils, we employed gene set enrichment analysis (GSEA) to explicit gene signatures of different subsets (Fig. 1e; Supplementary Tables S4–8). As a result, we defined 5 neutrophil subsets, including 3 conventional (cluster 0, cluster 1 and cluster 2) and 2 unconventional neutrophil subsets (cluster 3 and cluster 4). Conventional neutrophils were accordingly defined as inflammatory neutrophils (*S100P*^+^*S100A12*^+^), primed inflammatory neutrophils (*IL17RA*^+^*CXCL8*^+^), and reactive oxygen species (ROS)-responsive neutrophils (*HBA1*^+^*HBA2*^+^). These neutrophil subsets exhibited conventional neutrophil functions such as inflammation, chemotaxis and ROS response^24,25^. The other 2 subsets were defined as unconventional neutrophils, IFN-α-responsive neutrophils (*MX1*^+^*IFIT1*^+^*IFIT2*^*+*^) and T cell-regulatory neutrophils (*ANXA1*^+^*IL7R*^+^*CD52*^*+*^). In addition, Gene Ontology (GO) enrichment analysis with the signature genes of each subset was also performed to investigate the biological functions of these 5 subsets (Fig. 1f; Supplementary Tables S9–13). Similar to the results of GSEA, their enriched genes were involved in pathways, including inflammatory response (*S100A11*, *S100A12*, and *S100P*), regulation of defense response (*SOD2*, *CXCL8*, and *NCF1*), oxygen transport (*HBA1*, *HBA2*, *HBM*, and *HBB*), T cell differentiation (*ANXA1*, *CD74*, and *IL7R*) and response to IFN-α (*IFIT2*, *IFIT3*, and *IFITM1*), respectively. Taken together, human peripheral neutrophils were classified into conventional neutrophils and unconventional neutrophils by scRNA-seq analysis, and two neutrophil subsets with enhanced type I interferon signaling and T cell immune-regulatory functions were highlighted in our study.

We also performed joint analysis on female and male neutrophil datasets, and discovered significant sex-specific differences of the gene expression among different neutrophil subsets (Fig. 1g, h; Supplementary Tables S14 and S15). In females, higher expressed genes were observed associated with the regulation of lymphocyte function, such as T cell differentiation (*ANXA1*, *CD3D*, and *IL7R*) and T cell activation (*CD7*, *CD48*, *FCER1G*, and *HLA-A*) (Fig. 1g, h; Supplementary Tables S14 and S16). On the contrary, in males, neutrophil subsets were mainly involved in inflammatory responses, such as neutrophil degranulation (*S100A9*, *SLC2A3*, and *MMP25*), neutrophil extracellular trap formation (*NCF2*, *NCF4*, and *MAPK1*), positive regulation of innate immune response (*HLA-E*, *HLA-F*, *LAMP1*, and *SPI*) (Fig. 1g, h; Supplementary Tables S14 and S15). Increased expression of inflammatory (*S100A9*) and chemotaxis genes (*CCR1*, *CXCR4*, and *CXCL8*) was also identified in male conventional neutrophil subsets (Supplementary Fig. S1a). Enrichment analysis on the DEGs in conventional neutrophil subsets further confirmed the relatively enhanced leukocyte chemotaxis, inflammatory responses and responses to chemokine and external stimulus in males (Supplementary Fig. S1b). Taken together, sex-specific gene expression in neutrophils further illustrated that neutrophil-mediated inflammatory responses were more robust in males, whereas neutrophil-mediated T cell regulatory functions were relatively active in females. This prompted us a nuanced investigation into sex-specific neutrophil heterogeneity under homeostatic and auto-inflammatory condition.

### Sex-specific inter-cellular signaling among neutrophil subsets

Previous studies have focused on the cellular interaction between neutrophils and other immune cells^26^. The inter-cellular communications among neutrophil subsets remain elusive. Thus, we performed cellular connectomes analysis by Cellcall to explore sex-specific inter-cellular signaling among neutrophil subsets under homeostatic condition. Notably, T cell-regulatory neutrophil subset was the main signaling receiver from other neutrophil subsets with intensive signal number and strength (Fig. 1i, j), suggesting a pivotal effector role of T cell-regulatory neutrophils in inter-cellular communications among neutrophil subsets. And the inter-cellular signaling toward T cell-regulatory neutrophils, in both females and males, was mainly involved in TGFB1-TGFBR2 and CCL/CXCL-CXCR signaling (with a score larger than 0.5) (Fig. 1i, j). TGF-β was previously shown to be critical for the inhibition of naïve T cell differentiation and proliferation^27^. Unlike female neutrophils, TGFB1-TGFBR2 signaling was enhanced in males (Fig. 1i), indicating male-biased defects in T cell regulatory functions mediated by neutrophils. Moreover, CXCL1-CXCR2 and CXCL8-CXCR2 signalings were distinctly enhanced in males (Fig. 1i), as these two ligand–receptors have been well-established for their effects on mature/activated neutrophil mobilization and chemotaxis to the site of inflammation^28^. These findings confirmed our aforementioned observation that male neutrophils exhibited less T cell regulatory phenotype as well as more inflammatory and chemotaxis properties. Our study was consistent with previous investigations^3^ that neutrophil-mediated inflammation and infection exhibited male-biased morbidity, severity and mortality.

### Sex-specific alterations of neutrophil subsets in auto-inflammatory Behçet’s uveitis

**Fig. 2:**
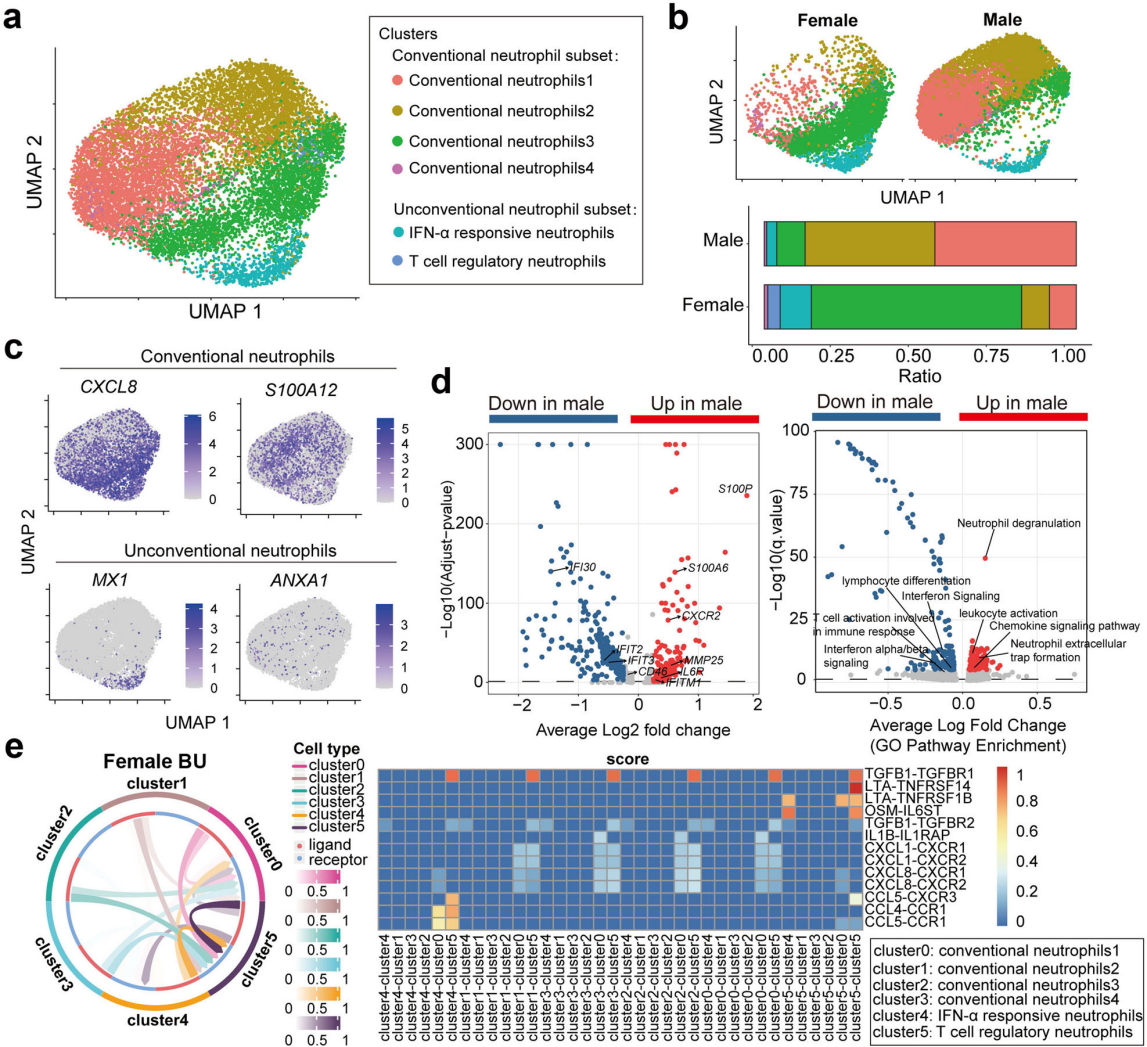
Sex-specific alterations of neutrophil subsets during BU pathogenesis. **a** UMAP visualization of 11,929 neutrophils from female (*n* = 8) and male BU patients (*n* = 10). **b** Upper panel: UMAP visualization of neutrophils from BU patients; lower panel: bar plot of the proportions of different neutrophil subsets shown in female and male BU patients, respectively. **c** UMAP plot overlays showing selected gene expression distribution. **d** Left panel: volcano plot showing the DEGs of neutrophils between female and male BU patients; right panel: GO enrichment of the DEGs. **e** Left panel: Circos plot of inter-cellular communication among different neutrophil subsets from female BU patients; right panel: heatmap showing the scores of different L-R pairs between neutrophil subsets. *n* refers to the number of each group.

Behçet’s disease is a systemic auto-inflammatory vasculitis, mainly affecting the small vessels mediated by neutrophils, and most commonly manifesting as mucosal and genital ulcers and uveitis^29^. About 3 out of 4 Behçet’s disease patients are with BU, which is more prevalent in men^30^. To further investigate the sex-specific alterations of neutrophil composition, we collected peripheral neutrophils from active female and male BU patients and profiled these neutrophils by scRNA-seq. A total of 11,929 neutrophils from female and male patients were partitioned into 6 clusters using two-dimensional UMAP (Fig. 2a). The proportions of these neutrophil subsets exhibited significant differences between female and male patients. The abundances of conventional inflammatory neutrophils (1 and 2) characterized by the higher expression of *S100A12* were significantly increased in male patients as compared with those in females (Fig. 2b, c; Supplementary Fig. S2a, b); nevertheless, remarkably attenuated *MX1*^+^ IFN-α-responsive neutrophil subset and a complete loss of *ANXA1*^+^ T cell-regulatory neutrophils were only observed in males under diseased condition (Fig. 2b, c).

In order to further explore the biological functions of these neutrophil subsets under diseased condition, GO enrichment analysis with the signature genes of each subset was performed (Supplementary Fig. S2c and Table. S17). Similar to the characteristic genes of neutrophil subsets under normal condition, conventional neutrophils 1–3 were defined as neutrophil extracellular traps (NETs)-producing neutrophils, inflammatory neutrophils and primed inflammatory neutrophils. The up-regulated genes in conventional neutrophil 4 were associated with aerobic respiration (*ATP5G1* and *ATP5G3*) (Supplementary Fig. S2a, b). Up-regulated genes in the other two clusters were uniquely enriched in response to IFN-α (*IFIT2*, *IFIT3*, *IFITM1*, and *IFITM3*), and the pathway of T cell activation (*ANXA1*, *CD7*, and *IL7R*) and T cell differentiation (*CD3D* and *CD28*) (Supplementary Fig. S2c).

Iterative analysis on DEGs was performed to evaluate the sex-specific differences of neutrophils under auto-inflammatory disease (Fig. 2d; Supplementary Table S18). Compared with female patients, up-regulated genes in males were mainly involved in neutrophil degranulation (*S100A11*, *S100P*, and *FCN1*), leukocyte activation (*IL6R*, *ITGAM*, and *SPI1*), chemokine signaling pathway (*CXCL1*, *HCK*, and *CXCR2*) and neutrophil extracellular trap formation (*FCGR2A*, *FCGR3A*, and *FCGR3B*), whereas genes such as *IFI30*, *IFIT1*, *IFIT2*, *IFITM1*, and *CD46*, involved in the IFN-α-responsiveness or regulation of T cell activation, were markedly downregulated (Fig. 2d; Supplementary Table S19). Notably, up-regulated genes in diseased female neutrophils were mainly involved in interferon (alpha/beta) signaling (*IFIT2*, *IFIT3*, *ISG15*, *IRF9*, and *IFITM2*) (Fig. 2d; Supplementary Table S20), which was distinctly different with healthy female neutrophils that were mainly involved in the regulation of T cell activation/differentiation (*ANXA1*, *CD3D*, *IL7R*, *CD7*, *CD48*, *FCER1G*, and *HLA-A*). Enrichment analysis of sex-specific DEGs within each subsets was also performed and its results showed that acute inflammatory response and neutrophil migration were enhanced in the conventional neutrophils from male patients, whereas the response to interferon-alpha in IFN-α-responsive neutrophil subset was relatively weak (Supplementary Fig. S2d). Our data indicated that enhanced interferon (alpha/beta) signaling in female neutrophils were more liable for efficient resolution of inflammatory responses in female patients, given that type I interferon signaling activates intra-cellular protective programmes and influences the development of innate and adaptive immune responses^31,32^. In addition, higher amount of unconventional neutrophils in females, including IFN-α-responsive and T cell-regulatory neutrophil subsets, also contributed to the efficient resolution of inflammatory responses. On the contrary, elevated conventional neutrophils were detected in male patients that exhibited exacerbated inflammatory response and NET production. NET formation is involved in the inflammatory process and participates in tissue damage^33^. In particular, NETs formation and markers of NETs levels are elevated in patients with Behçet’s disease and contributed to the pathophysiology of vasculitis^34,35^. These findings suggest a link between neutrophils and male-biased onset and progression of auto-inflammatory BU pathogenesis.

### Sex-specific alterations of inter-cellular signaling among neutrophil subsets in auto-inflammatory BU

In order to comprehensively understand the sex-specific interactions among different neutrophil subsets during BU, we analyzed the inter-cellular signaling among diseased neutrophil subsets by Cellcall. Inter-cellular signalings among neutrophil subsets were not detected in male patients due to the complete loss of T cell regulatory subset. Unexpectedly, in addition to T cell-regulatory neutrophil subset that was detected as an exclusive receiver in healthy female neutrophils, the inflammatory neutrophil subset was also detected as a prominent receiver of signaling from other neutrophil subsets (Fig. 2e). Under diseased conditions, inter-cellular signaling incoming toward inflammatory neutrophils were mainly involved in CCL-CXCR/CCR and IL1B-IL1RAP. CCL-CXCR/CCR signaling is rapidly induced upon inflammation, and subsequently promotes the recruitment of leukocytes, including neutrophils, T cells and macrophages^36,37^, and IL-1β is an important inflammatory interleukin that is synthesized and secreted by activated neutrophils^38^. Thus, above data of inter-cellular signaling among neutrophil subsets, further indicated that female diseased neutrophils were more prone to inflammation and subsequently inflammatory neutrophil cascade, as compared with healthy female neutrophils. In addition, more inter-cellular signaling incoming towards T cell-regulatory neutrophils was detected in female BU patients, such as LTA-TNFRSF, as compared with female healthy neutrophils. TNFRSF molecules, including TNFRSF14 and TNFRSFB, are essential for communication between immune cells, and their dysfunction can lead to the development of autoimmune diseases^39^. Besides TNFRSF molecules, TGFB1-TGFBR1 signaling was also detected enhanced in female BU neutrophils (Fig. 2e). Several studies have reported that TGFB1 is critical to the maintenance of immune homeostasis as deletion of TGFB1 results in a generalized inflammatory syndrome and autoimmunity^40^. Taken together, these data provide more evidence and explain, at least partially from neutrophils’ perspective, why females are more prone to develop autoimmune disease, and why they have better ability at self-resolving under auto-inflammatory BU.

### Sex-specific neutrophil magnitude correlated with clinical manifestations

**Fig. 3:**
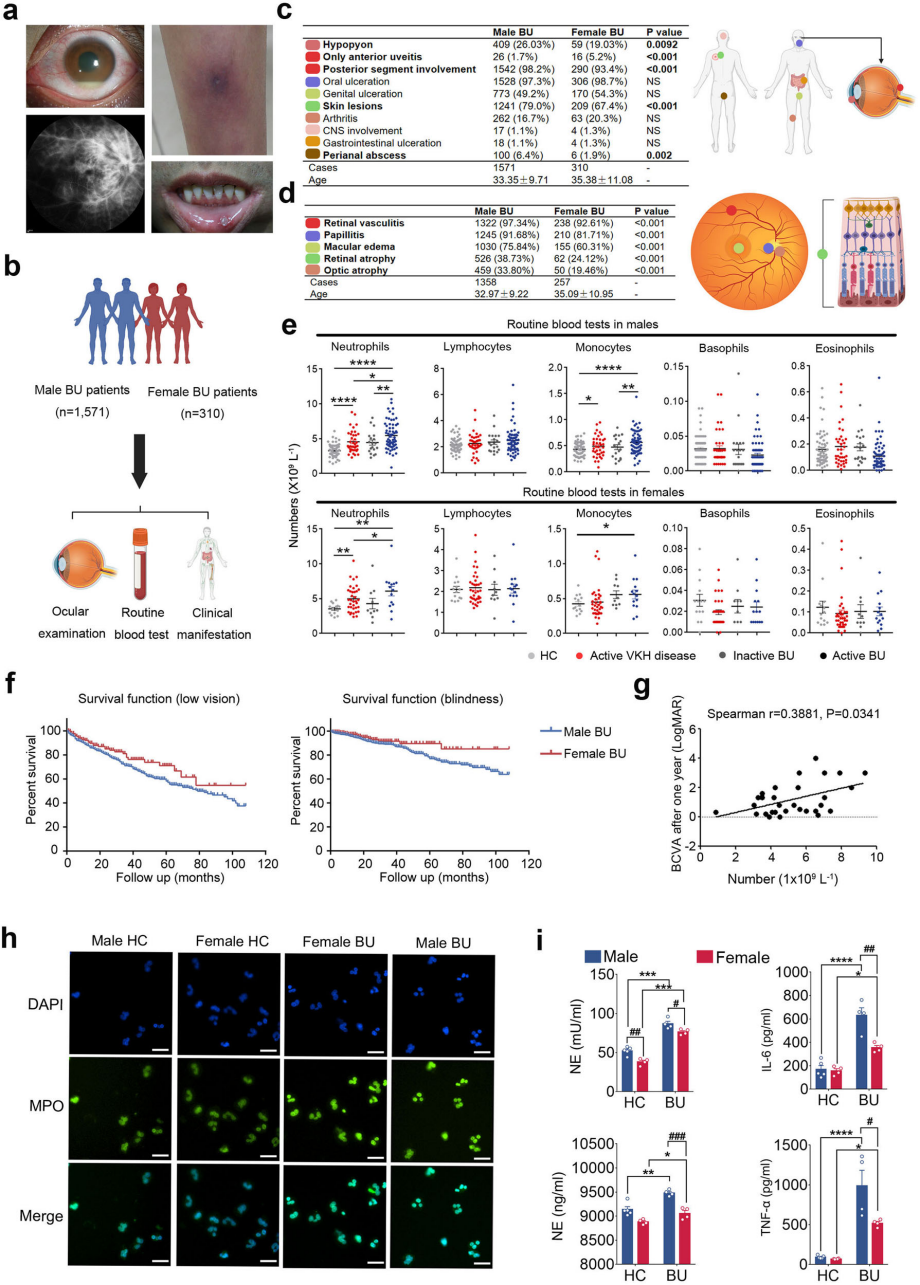
Retrospective study identifies neutrophil magnitude correlated with sex-specific clinical manifestations. **a** Photograph showing the BU manifestations, including hypopyon, skin lesions and oral ulceration, as well as FFA showing diffuse leakage from optic and vessels. **b** Schematic representation of the experimental design for retrospective analysis. **c** Incidences of systemic manifestations in female (*n* = 310) and male (*n* = 1571) patients. Data were analyzed by the Pearsons χ^2^ test. **d** Incidences of ocular manifestations in female (*n* = 257) and male (*n* = 1358) patients. Data were analyzed by the Pearsons χ^2^ test. **e** The number of immune cells in females and males among active BU patients (female: *n* = 14; male: *n* = 64), inactive BU patients (female: *n* = 10; male: *n* = 19), active VKH disease patients (female: *n* = 36; male: *n* = 38) and HCs (female: *n* = 14; male: *n* = 64), respectively. **f** Kaplan-Meier curves of cumulative survival of patients with low vision (0.05 ≤ visual acuity < 0.3) and blindness (visual acuity < 0.05) in female BU patients (*n* = 197) vs male BU patients (*n* = 901). **g** Correlation analysis between neutrophil number at first visit and visual acuity (LogMAR) after one-year treatment (female: *n* = 8; male: *n* = 28). **h** Confocal microscopy showing the MPO of neutrophils from active BU patients and HCs. Scale bars, 20 μm. **i** Sex-specific differences in the secretion of NETs and pro-inflammatory cytokines by peripheral neutrophils from active BU patients (female: *n* = 4; male: *n* = 4) and HCs (female: *n* = 4; male: *n* = 5). Data are shown as means ± SEM. *P < 0.05; **P < 0.01; ***P < 0.001; ****P < 0.0001. ^#^*P* < 0.05; ^##^*P* < 0.01; ^###^*P* < 0.001; *P*-values were calculated by the one-way ANOVA (Tukey’s multiple comparison test). In all instances, *n* refers to the number of each group.

Emerging investigations reported the differences between auto-inflammatory and autoimmune diseases, as well as the pathogenesis and mechanisms of female-biased autoimmune diseases. Zoltán et al. have demonstrated that classical auto-inflammatory conditions are characterized by a predominance of innate immunity and have no sex dominance^41^. However, higher prevalence and more severe manifestations of BU were observed in men^42^. Thus, we first performed a retrospective study on 1881 BU patients, including 310 females and 1571 males, to assess the direct correlation between neutrophils and clinical manifestations (Fig. 3a, b). BU was diagnosed according to the International Criteria for Behçet’s disease (ICBD)^43^. All the BU patients were performed a comprehensive ophthalmic examination, including best-corrected visual acuity (BCVA), slit-lamp bio-microscopy, and ophthalmoscopy through a dilated pupil, as well as systemic assessment including anogenital and dermatologic history, abnormalities in the central nervous system (CNS) and gastrointestinal disorders, routine blood test, liver and kidney function test. Consistent with our previous investigations^15^, higher incidence of posterior segment (eye) involvement (98.2%), skin lesions (79.0%) and perianal abscess (6.4%), as well as a lower rate of only anterior segment (eye) involvement were observed in male BU patients (Fig. 3c), and the clinical manifestations of posterior segment involvement also exhibited pronounced male-biased morbidity (Fig. 3d). In detail, 1615 patients (257 females, 1358 males) were assessed with fundus fluorescein angiography (FFA) and optical coherence tomography (OCT). Higher incidence of posterior segment involvement in male patients, such as retinal vasculitis (97.34%), papillitis (91.68%), macular edema (75.84%), retinal atrophy (38.73%) and optic atrophy (33.80%), was further determined (Fig. 3d). These data provide direct evidence that male patients exhibit distinctly more severe manifestations, especially eye involvement with uveal which is rich in vasculature. These male-biased clinical features led us to further explore sex-specific abnormalities of the circulating neutrophils.

In order to explore circulating neutrophil responses under auto-inflammatory disease, we analyzed two cohorts of patients, one with BU and the other one with Vogt-Koyanagi-Harada (VKH) disease. Both of them are systemic inflammatory diseases with eye involvement but have different immune responses. VKH disease was diagnosed as a revised criteria finalized by an international committee and modified criteria developed by our team^44,45^. Neither BU nor VKH disease patients involved in this study received any treatments within at least 2 weeks before sampling to capture a pharmacologically unperturbed landscape. To deduce the immune pathogenesis of BU patients, we chose patients with active VKH disease as well as healthy females and males as control subjects. According to the retrospective analysis of all the medical records, including active BU patients (*n* = 78) and inactive BU patients (*n* = 29), VKH disease patients (*n* = 74), and healthy controls (HCs) (*n* = 78), we discovered increased numbers of both neutrophils and monocytes in both active female and male BU patients as compared with HCs, whereas there was only an increased neutrophil number observed in active VKH disease patients (Fig. 3e). Importantly, a significantly higher number of neutrophils was observed in active BU patients (both females and males) as compared with active VKH disease patients (both females and males). Moreover, male active BU patients always exhibited an increased number of neutrophils as compared with inactive patients, whereas this difference was not detected in females. These results were consistent with previous investigations that elevated neutrophil numbers and excessive neutrophil responses were detected in the circulation of Behçet’s disease patients^46,47^, and our data further illustrated the male-biased exacerbated neutrophil responses in male BU patients.

We also evaluated the sex-specific difference in the visual prognosis of BU patients and its direct correlation with neutrophil amounts. Kaplan-Meier survival analysis was performed to compare the risks of low vision and blindness at 5 and 9 years between female and male BU patients, respectively. The risk of low vision in females/males was 28.5% vs 41.8% at 5 years, and 45.3% vs 62.5% at 9 years (log-rank = 4.304; *P* = 0.038) (Fig. 3f). Accordingly, the risk of blindness in females/males was 10.5% vs 23.1% at 5 years, and 14.9% vs 36.2% at 9 years, respectively (log-rank = 4.043; *P* = 0.044). Thus, a positive correlation between neutrophil number (at the first visit) and BCVA (LogMar) 1 year after treatment in male patients (Fig. 3g) further demonstrated the important role of neutrophils in the outcome of BU, although no such correlation was observed in female patients.

### Sex-specific aberrant neutrophil response and Th17–Treg cell imbalance in BU patients

In order to confirm our observations by scRNA-seq, blood samples from active BU patients (females and males) and HCs (females and males) were collected respectively. Given the close relation between NETs production and Behçet’s disease severity^34^, there is a need to further determine the sex-specific NETosis of circulating neutrophils during BU pathogenesis. The NETosis of circulating neutrophils was evaluated by assessing myeloperxidase (MPO) and neutrophil elastase (NE) expression^48^. Here, elevated NET formation was detected in neutrophils collected from male active BU patients, as compared with HCs or female BU patients (Fig. 3h, i). Activated neutrophils can up-regulated the production of pro-inflammatory cytokines, such as IL-6 and TNF-α^24^. Thus, we further examined the production of IL-6 and TNF-α in the supernatant of neutrophils to assess neutophils activity and found that protein levels of IL-6 and TNF-α were significantly increased in the supernatants of BU neutrophils, especially in male BU neutrophils (Fig. 3i). Moreover, we also detected apoptosis of neutrophils to evaluate the state of inflammation, as delayed apoptosis of neutrophils always leads to lasting and exacerbated inflammatory responses^49^. As expected, reduced apoptotic neutrophils was detectd in male BU patients, as compared with either female BU patients or healthy controls (Supplementary Fig. S3a). These observations demonstrated excessive neutrophil activation and exacerbated inflammatory response in male BU patients.

The imbalance between Th1/Th17 and Treg cells is a critical biological event during BU pathogenesis^50^. Thus, we further analyzed the sex-specific differences in frequencies of Th1, Th17 and Treg cells collected from BU patients by flow cytometry, respectively. As expected, a marked elevated percentage of CD4^+^ T cells expressing either IFN-γ or IL-17A was detected in BU patients, especially in males, and the total number of Treg cells was substantially diminished as compared with HCs (Supplementary Fig. S3b), indicating sex-specific disruption of Th17–Treg cell balance during BU pathogenesis. These laboratory data presented above further confirmed male-biased dysregulated neutrophil responses, as well as Th17–Treg cell imbalance in BU patients, which was consistent with aforementioned scRNA-seq data, and also suggested their associations with more severe clinical manifestations and poor prognosis we reported in male BU patients. Taken together, the aforementioned results led us to investigate the sex-specific association between the heterogeneity of neutrophil composition and the immune pathogenesis, as well as long-term prognosis of auto-inflammatory BU.

### Sex-specific alterations of neutrophils during auto-inflammatory BU pathogenesis

Iterative analysis was performed to describe a complete atlas of sex-specific neutrophil composition alterations under both auto-inflammatory and homeostatic condition. Neutrophils from females and males were partitioned into 7 and 6 clusters using two-dimensional UMAP, respectively (Supplementary Fig. S4a, e). For conventional neutrophil compartments (Supplementary Fig. S4b, f and Tables S21 and S25), a significantly increased abundance of inflammatory neutrophils (cluster 0 in females and males) was observed in both female and male patients (Supplementary Fig. S4c, g). In addition, a significantly decreased proportion of ROS-responsive neutrophils (Supplementary Fig. S4c, g) was detected in BU patients as compared with HCs, indicating decreased oxidative stress-induced neutrophil apoptosis^51^. These results were consistent with our aforementioned observations that a significant elevation of circulating inflammatory neutrophils with a lower frequency of apoptotic cells was detected in BU patients. On the contrary, for the unconventional neutrophil compartments (Supplementary Fig. S4b, f and Tables S21 and S25), the proportions of IFN-α-responsive neutrophils and T cell-regulatory neutrophils were decreased (Supplementary Fig. S4c, g). Flow cytometry analysis further confirmed these sex-specific alterations in unconventional neutrophils during BU pathogenesis (Supplementary Fig. S5a, b).

We also explored the sex-specific differences of DEGs among these diseased neutrophils. The results of GO analysis revealed that up-regulated DEGs in diseased neutrophils were mainly involved in pathways of inflammatory responses (*IL1B*, *IL17RA*, *S100A8* and *S100A12*) and leukocyte chemotaxis (*CXCR1*, *CXCR2* and *CXCL8*) (Supplementary Fig. S4d, h and Tables S22–24, S26–28). In male BU patients, up-regulated genes of neutrophils were mainly involved in neutrophil extracellular trap formation (*ITGB2* and *IL1B*) as compared with male HCs, and of note, genes that contributed to interferon-alpha/beta signaling (*ISG15*, *IFIT2*, *IFIT3* and *STAT1*) were significantly downregulated (Supplementary Fig. S4d and Tables S22–24). These results of sex-specific gene expression in neutrophils suggested male-specific exacerbated neutrophil-mediated inflammatory response and attenuated interferon type I signaling pathway during auto-inflammatory BU. In females, the interferon type I signaling pathway in neutrophils was not affected under diseased condition, though the gene expression associated with the regulation of T cell differentiation/activation was significantly decreased (Supplementary Fig. S4h and Tables S26–28). Taken together, these results demonstrated higher proportions and enhanced function of unconventional neutrophils in females under both homeostatic and auto-inflammatory conditions, as compared with males.

### Pseudotime analysis reveals the cell fate transition of neutrophil subsets

**Fig. 4:**
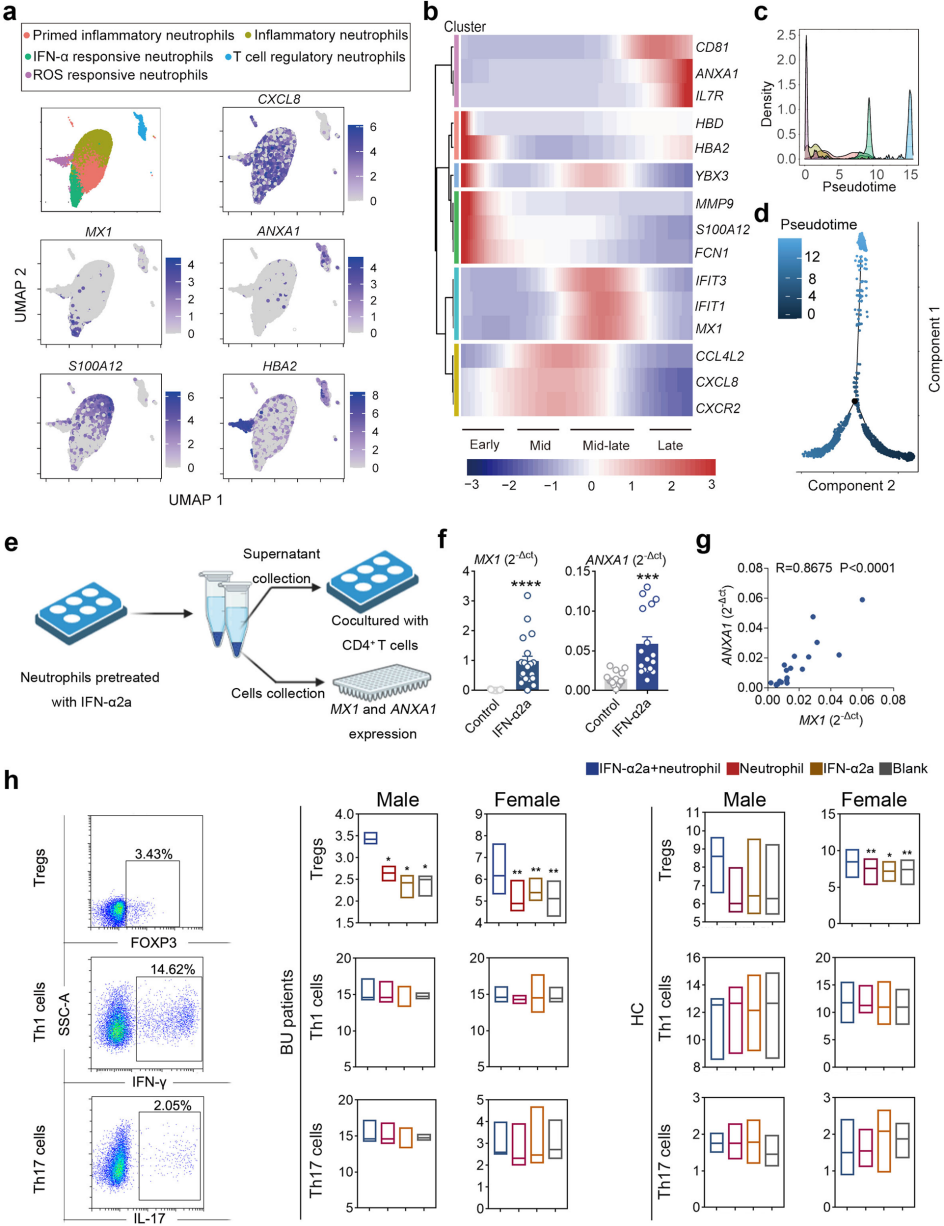
IFN-α2a renders human neutrophil more immune-regulatory phenotype. **a** UMAP visualization of 17,270 neutrophils from BU patients (*n* = 18) and HCs (*n* = 16). **b** Heatmap showing relative expression of genes significantly (FDR < 0.05) covaried with pseudotime. **c** Cluster distribution density along pseudotime. **d** Trajectory analysis of peripheral neutrophils captures a pseudotime progression. **e** Schematic representation of the experimental design for co-culture experiments. **f** mRNA expression of *MX1* and *ANXA1* in the peripheral neutrophils from healthy individuals (*n* = 17) after treatment with/without IFN-α2a. **g** Correlation of *MX1* and *ANXA1* mRNA expression of peripheral neutrophils from healthy individuals (*n* = 17). **h** Effects of IFN-α2a pre-treated neutrophils on the frequencies of Th1/Th17 cells and Tregs in CD4^+^ T cells from active BU patients (female: *n* = *4*; male *n* = *3*) and healthy individuals (female: *n* = *5*; male: *n* = *4*). Data are shown as means ± SEM. *P < 0.05; **P < 0.01; ***P < 0.001; ****P < 0.0001. *P*-values were calculated by the Wilcoxon matched pairs test. In all instances, *n* refers to the number of each group.

We partitioned all the collected neutrophils into 5 clusters and then performed a pseudotime analysis to determine cell fate transition trajectory among them (Fig. 4a). According to the gene signatures (Supplementary Table S29), we observed a trajectory that progressed from ROS-responsive and inflammatory neutrophils, then to primed inflammatory neutrophils and IFN-α-responsive neutrophils, eventually culminated with T cell-regulatory neutrophils (Fig. 4b–d). To further determine the biological processes driving pseudotime components, covaried genes along with pseudotime were assessed. We clustered all covaried genes along with pseudotime (false discovery rate: FDR < 0.05), and defined four phases as (i) early, (ii and iii) mid/mid-late and (iv) late according to the gene expression. At early phase, marker genes of ROS-responsive neutrophil subsets and inflammatory neutrophil subsets, such as *HBD*, *YBX3*, *MMP9*, and *S100A12*, were detected. In the mid and mid-late phases, genes involving chemotaxis regulation (*CCL4L2*, *CXCL8*, and *CXCR2*) and response to IFN-α (*IFIT1*, *IFIT3*, and *MX1*) were detected, respectively. Of note, gene expression that associated with the regulation of T cell activation (*CD81*, *ANXA1*, and *IL7R*), namely T cell-regulatory neutrophil subset, was markedly elevated at late phase, whereas gene expression involved in inflammatory response, chemotaxis regulation, and response to IFN-α gradually attenuated and disappeared, as well as response to ROS. Wright-Giemsa and immunofluorescence staining was performed on sorted human Annexin A1^+^ neutrophils, and classical polymophor-nuclei was observed, indicative of a mature neutrophil phenotype (Supplementary Fig. S6a, b).

These data suggested a distinct trajectory of the cell fate transition among unconventional neutrophil subsets, which was from IFN-α-responsive neutrophil subset to T cell-regulatory neutrophil subset, and further indicated the final effector role of T cell-regulatory neutrophil subsets.

### IFN-α2a renders human neutrophil more immune-regulatory phenotype

Traditional therapeutic approaches (using local and systemic immunomodulatory and immunosuppressive drugs) for treating auto-inflammatory disease have limited efficacy and serious side effects^52^. Emerging investigations reported IFN-α2a offering moderate benefits for treating auto-inflammatory Behçet’s disease, especially severe and relapsing uveitis^53,54^. Several clinical trials discovered that the outcomes and efficacy of IFN-α2a for BU are circumstantial and, in most cases, sex-specific^55^. Therefore, we first investigated the difference in the response of neutrophils to IFN-α between female and male patients, and found that male patients had lower mRNA expression levels of IFN-α-responsive genes (*IFIT1*, *IFIT3*, and *IFI44L*) in IFN-α2a-treated neutrophils as compared with female patients (Supplementary Fig. S7a). The cell fate transition between two unconventional neutrophil subsets demonstrated by pseudotime analysis prompted us to ask if IFN-α2a treatment was able to promote the differentiation of early/middle-phase subsets to T cell regulatory subset. To further assess the T cell regulatory function of IFN-α2a-treated neutrophils, we pre-treated neutrophils collected from BU patients and healthy donors with IFN-α2a, and then cocultured with CD4^+^ T cells, respectively (Fig. 4e). We first treated human neutrophils with IFN-α2a and subsequently assessed neutrophil phenotype. The gene expression of *MX1* and *ANXA1* in neutrophils was significantly upregulated (Fig. 4f). More importantly, *MX1* expression was positively correlated with the expression of *ANXA1* in neutrophils (Fig. 4g). We also assessed the T cells and supernatants collected after coculturing with IFN-α2a-treated healthy and diseased neutrophils, respectively. A marked expansion of Treg cells, accompanied with increased production of regulatory cytokine IL-10, was observed in IFN-α2a pre-treated neutrophil group, and the expansion was more pronounced in female neutrophils pre-treated group. However, there was no effect observed on the total number of IFN-γ- or IL-17A-expressing CD4^+^ T cells, as well as their effector cytokines (IFN-γ and IL-17) (Fig. 4h; Supplementary Fig. S7b). CSFE staining assay was also performed to evaluate T cell proliferation. However, the data collected showed that IFN-α2a-pre-treated neutrophil had no effect on T cell proliferation (Supplementary Fig. S7c). These results demonstrated the effects of IFN-α2a on neutrophil phenotype shift that further resulted in Treg cell expansion, indicating the important role of neutrophil subsets in maintaining Th17–Treg cell balance during BU pathogenesis.

### T cell-regulatory neutrophils exhibit immune-regulatory property

Our scRNA-seq data above, accompanied with in vitro co-culture experiments pointed to the regulatory effects of T cell-regulatory neutrophil subset. Given the complete loss of this subset in male BU patients, we next focused on this subset and its function. We first leveraged a published scRNA-seq dataset (GSE137540) of murine-derived neutrophils to confirm the existence of similar neutrophil subsets in mice as in human circulation^56^. Here, neutrophils from mice were partitioned into 5 clusters using two-dimensional UMAP (Supplementary Fig. S8a, b). According to the gene signatures (Supplementary Fig. S8c and Table S30), a similar neutrophil subset–T cell-regulatory neutrophils (cluster 4; *Anxa1*^+^) was observed in mice (Supplementary Fig. S8c).

We next employed a commonly used murine model, experimental autoimmune uveitis (EAU), which is relatively more suitable for the study of BU among animal models for Behçet’s disease^57^, to identify the in vivo functions of T cell-regulatory neutrophils. In detail, C57BL/6 J mice were immunized with human retinal interphotoreceptor retinoid-binding protein (IRBP) 651–670 peptide. After immunization, the frequencies of splenic Th1, Th17, and Treg cells as well as neurophils during different phases of EAU were detected by flow cytometry (Supplementary Fig. S9). As expected, elevated frequencies of Th1 and Th17 cells were observed in both female and male mice after immunization, accompanied with abrogated Treg cells (Supplementary Fig. S10a, b). These results were consistent with previous investigations^58^, and the disease model was successfully established. In addition, we observed that female mice exhibited early (2 weeks) Th1/Th17 cell responses as well as Treg cell diminishment, while male mice displayed an exacerbated Th17–Treg cell imbalance at recovery phase (4 weeks) after immunization (Supplementary Fig. S10a, b). Higher proportions of pan neutrophils and Annexin A1^+^ T cell-regulatory neutrophil subset were also observed in female mice throughout the immunization as compared with male mice (Supplementary Fig. S8d), which was consistent with aforementioned scRNA-seq data of neutrophils collected from human BU patients. Importantly, we noticed that the frequency of Annexin A1^+^ neutrophils was positively correlated with Treg cells, and negatively correlated with Th1/Th17 cells in female mice, however, no such correlation was observed in male mice (Supplementary Fig. S10c). Taken together, these data collected from murine model, further corroborated our findings that neutrophil subset with T cell regulatory feature was more pronounced in females under auto-inflammatory disease, with function of Treg cell amplification and Th1/Th17 cell attenuation.

**Fig. 5:**
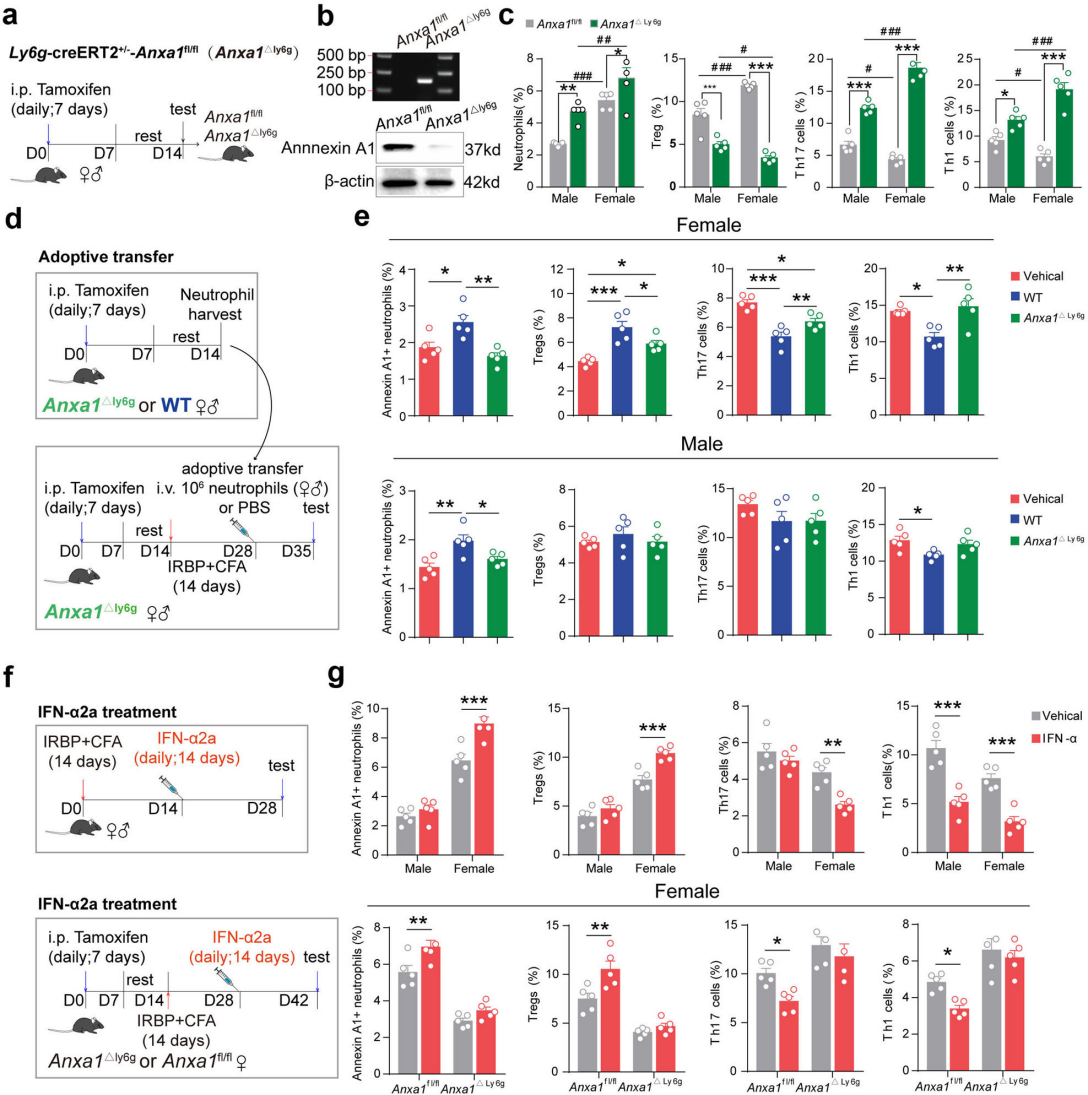
Annexin A1^+^ neutrophils determine the Th17–Treg cell balance and immunoregulatory effect of IFN-α2a in vivo. **a** The experimental scheme of the creation of *Anxa1*^ΔLy6g^ mice. **b** Upper panel: PCR genotyping of *Anxa1*^fl/fl^ and *Anxa1*^ΔLy6g^ mice; lower panel: the protein level of Annexin A1 in the neutrophils from *Anxa1*^fl/fl^ and *Anxa1*^ΔLy6g^ mice. **c** Frequencies of neutrophils, Th1/Th17 cells and Tregs in *Anxa1*^fl/fl^ mice with EAU and *Anxa1*^ΔLy6g^ mice with EAU, respectively. *n* = 5 per group. **d** The experimental scheme of adoptive transfer experiment. **e** Frequencies of Th1/Th17 cells and Tregs, as well as the frequency of Annexin A1^+^ neutrophils in *Anxa1*^ΔLy6g^ EAU mice after adoptive transfer of neutrophils from normal WT mice and *Anxa1*^ΔLy6g^ EAU mice, respectively. *n* = 5 per group. **f** The experimental scheme of IFN-α2a treatment on *Anxa1*^fl/fl^ EAU, and *Anxa1*^ΔLy6g^ EAU mice. **g** Effects of IFN-α2a on the T cells and Annexin A1^+^ neutrophils in *Anxa1*^fl/fl^ EAU mice, and *Anxa1*^ΔLy6g^ EAU mice. *n* = 5 per group. Data are shown as means ± SEM. *P < 0.05; **P < 0.01; ***P < 0.001. ^#^*P* < 0.05; ^##^*P* < 0.01; ^###^*P* < 0.001; *P*-values were calculated by the Mann-Whitney U test. In all instances, *n* refers to the number of each group.

To provide conclusive in vivo evidence for T cell regulatory activity of Annexin A1^+^ neutrophils, we generated mice with targeted deletion of the *Anxa1* gene in neutrophils by crossing *Anxa1*^fl/fl^ mice with *Ly6g*^Cre/+^ mice (*Ly6g*^Cre/+^, *Anxa1*^fl/fl^, or *Anxa1*^ΔLy6g^) (Fig. 5a). As expected, the expression of Annexin A1^+^ in neutrophils was substantially diminished in *Anxa1*^Δ^^Ly6g^ mice (Fig. 5b), and subsequently Treg cell expansion was significantly impaired after immunization, accompanied with elevated frequency of IFN-γ or IL-17A expressing CD4^+^ T cells (Fig. 5c). These data confirmed the important role of Annexin A1^+^ neutrophils in maintaining Th17–Treg cell balance. To further confirm the T cell regulatory function of Annexin A1^+^ neutrophils in vivo, adoptive transfer experiments were performed in both female and male mice, respectively (Fig. 5d). Splenic neutrophils isolated from wildtype (WT) mice or *Anxa1*^ΔLy6g^ mice were injected into immunized *Anxa1*^ΔLy6g^ mice with same genders, respectively. In female mice, an increased abundance of Annexin A1^+^ neutrophils and subsequently restored Th17–Treg cell balance were observed in immunized *Anxa1*^ΔLy6g^ mice after receiving neutrophils from WT mice as compared with those receiving neutrophils from *Anxa1*^ΔLy6g^ mice (Fig. 5e). However, although slightly increased Annexin A1^+^ neutrophils were detected in male immunized *Anxa1*^ΔLy6g^ mice, the Th17–Treg cell imbalance was not recovered after adoptive transfer (Fig. 5e). These findings combined with the in vitro data collected from BU patients firmly established that the T cell regulatory activity of neutrophils could be attributed, at least in part, to Annexin A1^+^ neutrophils, and were linked to BU progression via regulating Th17–Treg cell imbalance. In addition, Annexin A1^+^ neutrophils exhibited a female-specific immune-regulatory effect on Th17–Treg cell balance in vivo under both healthy and immunized conditions in murine model.

### IFN-α2a renders neutrophils more immune-regulatory phenotype female-specifically in vivo

We next attempted to explore the effect of IFN-α2a on neutrophils in vivo (Fig. 5f). Consistent with above in vitro data, after administration of IFN-α2a to immunized mice, the unique responsiveness of Annexin A1^+^ neutrophils and Treg cells was also detected in female mice in vivo, and accompanied with significantly decreased Th17 cells as well as clinical and histopathological scores (Fig. 5g and Supplementary Fig. S11a, b). Correspondingly, the protein levels of IL-17 and IFN-γ in the serum of EAU were decreased after administration of IFN-α2a, and the protein level of IL-10 was increased (Supplementary Fig. S11c). However, in male mice, although IFN-α2a treatment induced slightly increased Annexin A1^+^ neutrophils accompanied with decreased Th17 cells, there was no statistically significant difference observed (Fig. 5g). These data collected from male mice were inconsistent with in vitro studies that IFN-α2a pre-treated neutrophils from male humans, which was able to induce the expansion of Treg cells. It was assumed that the inconsistency was primarily owing to differences between species and more immune cell types participating the immune responses in vivo. Thus, in order to confirm the female-specific actions of IFN-α2a on Annexin A1^+^ neutrophil subset in vivo, immunized female *Anxa1*^ΔLy6g^ mice and their littermates were treated with IFN-α2a, respectively. As expected, IFN-α2a treatment expanded Annexin A1^+^ neutrophils in female *Anxa1*^fl/fl^ mice, accompanied with enhanced Treg cell abundance as well as suppressed Th1 and Th17 cell responses (Fig. 5g). However, deletion of Annexin A1^+^ neutrophils in mice eliminated the regulatory effects of IFN-α2a treatment on the frequencies of Th1/Th17 cells and Treg cells (Fig. 5g). To sum up, our data suggested that IFN-α2a rendered neutrophils a more immune-regulatory phenotype via Annexin A1 signaling female-specifically to regulate Th17–Treg cell balance and maintain homeostasis.

### Unique regulatory pathways control neutrophil subsets phenotype during BU pathogenesis

**Fig. 6:**
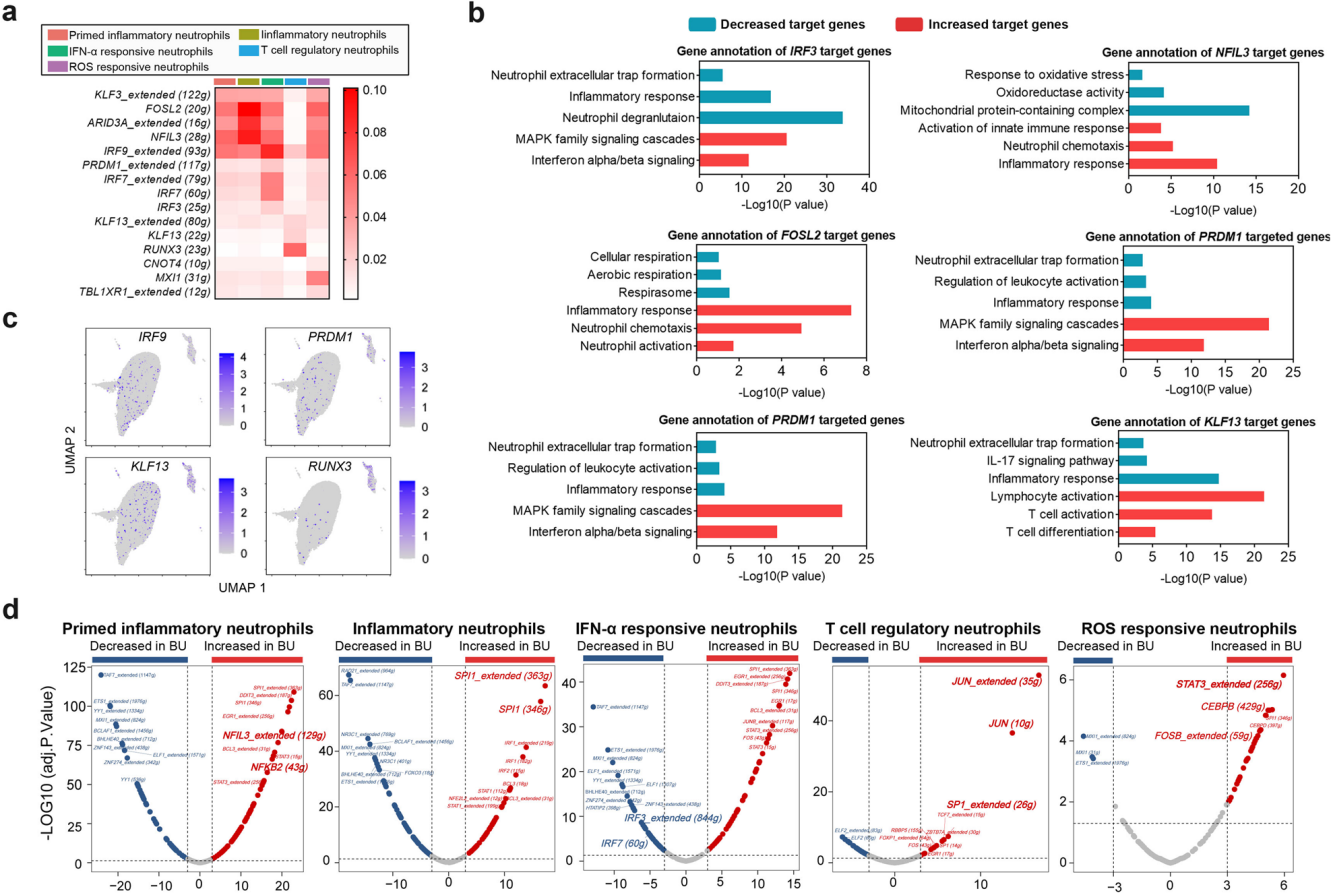
Unique regulatory pathways control neutrophil subsets phenotype during BU pathogenesis. **a** Heatmap showing the mean expression of TFs in each neutrophil subset (cells from BU patients: *n* = 18; HCs: *n* = 16). **b** GO analysis of TFs’ target genes; red: increased target genes; green: decreased target genes. **c** UMAP plot overlays showing selected TF expression distribution across clusters. **d** Volcano plot showing the differentially expressed TFs (adjusted *P*-value < 0.05) in the different cluster subsets between BU patients and HCs. In all instances, *n* refers to the number of each group.

IFN-α responding signaling of neutrophils, as well as Annexin A1 pathway, have not been routinely considered during inflammatory response, since neutrophils are generally considered to be homogenous with a uniform pro-inflammatory capacity. A recent study by Sarthak et al. performed RNA-seq analysis on healthy individuals and revealed a subset of neutrophils with IFN-gene signature and its abundance was increased in females^21^. However, further exploration on the phenotypic characteristics or functionality of this subset is still needed, especially under pathological state. In order to uncover the phenotype shifting of these two unconventional neutrophil subsets during BU pathogenesis, further exploration into factors driving their phenotypes alteration was carried out. Gene regulatory network reconstruction by single-cell regulatory network inference and clustering (SCENIC) was employed to identify differentially activated transcription factors (TFs) of the target genes in diverse neutrophil subsets. DEGs and TFs were analyzed and compared between conventional and unconventional neutrophils. Here we discovered, in conventional inflammatory neutrophils, activation of *FOSL2* and *NFIL3* was mostly pronounced, and their target genes encoding chemotactic and inflammatory proteins, such as *CXCR2*, *S100A12*, *NCF4*, and *NCF1*, contributed to neutrophil chemotaxis, inflammatory response, and neutrophil extracellular trap formation (Fig. 6a–c). These data confirmed the traditional pro-inflammatory features of inflammatory neutrophils.

We then focused on the differentially activated TFs in unconventional neutrophil subsets (Fig. 6a–c). The activation of *IRF3*, *IRF7* and *PRDM1* was identified as the hallmark of IFN-α-responsive neutrophils and predicted to drive *ISG15*, *MX1*, and *IFIT1* gene expression. Notably, *IRF3* and *IRF7* expression in IFN-α-responsive neutrophils was significantly attenuated in BU patients as compared with HCs (Fig. 6d). These impaired TFs in IFN-α-responsive neutrophils accordingly down-regulated the gene expression of IFN-α-responsive elements (*ISG15*, *MX1*, and *IFIT1*) during BU pathogenesis. Notably, *RUNX3* and *KLF13* were uniquely identified as active TFs in Annexin A1^+^ neutrophils (Fig. 6a–c). Intriguingly, a subset of the *RUNX3* and *KLF13* targetome was associated with the pathway of regulating lymphocyte activation and differentiation, which was consistent with the gene signature observed in Annexin A1^+^ neutrophils (Fig. 6b). In contrast, the expression of target genes that were associated with inflammatory responses and neutrophil extracellular trap formation, was attenuated in Annexin A1^+^ neutrophils (Fig. 6b). Stratified analysis by sex showed that the expression levels of *NFIL3*, *SPI1* and *FOSL2* were increased in the inflammatory neutrophils and chemotactic neutrophils from healthy males as compared to healthy females, whereas the expression of *IRF3* and *JUN* was decreased in the IFN-α-responsive neutrophils and T cell-regulatory neutrophils, respectively, For BU patients, there were similar trends in the *NFIL3*, *SPI1* and *FOSL2* expression of male inflammatory neutrophils and chemotactic neutrophils as well as the *MXI1* expression of male IFN-α-responsive neutrophils (Supplementary Fig. S12a, b). To summarize, the feature of neutrophil subsets was altered by unique and sex-specific regulatory pathways during BU pathogenesis. Opposite activation patterns were observed in conventional inflammatory neutrophils and unconventional neutrophils, further illustrating the distinct unconventional neutrophil subsets with different functions during BU pathogenesis.

### Male BU-specific single nucleotide polymorphisms impair unconventional neutrophil subsets

**Fig. 7:**
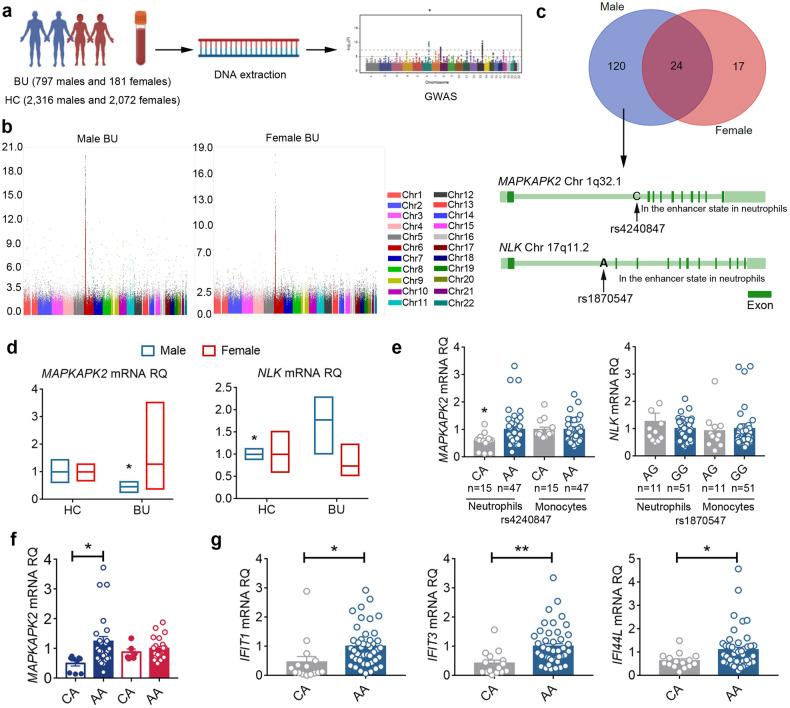
GWAS identifies male-specific risk SNPs contributing to the deficiency of IFN-α-responsive neutrophils. **a** The experimental scheme of sex-specific stratification analysis on GWAS. **b** Manhattan plot of *P* values on the –log10 scale for 753,745 genotyped SNPs in the GWAS (female BU: *n* = 181; male BU: *n* = 797; female HCs: *n* = 2072; male HCs: *n* = 2316). SNP locations are from Build 37/hg19. **c** Left panel: Rs4240847 and rs1870547 are in the enhancer state of *MAPKAPK2* and *NLK* of neutrophil, respectively; right panel: specific and shared disease-related genes between male and female BU patients. **d** Sex-specific differences in the *MAPKAPK2* and *NLK* expression by neutrophils between active BU patients and HCs (female BU *n* = 4; male BU *n* = 5; female HCs *n* = 4; male HCs *n* = 4). **e** The relation between *MAPKAPK2* SNP rs4240847 genotype (CA: *n* = 15; AA: *n* = 47) and *MAPKAPK2* mRNA expression, as well as the relation between *NLK* SNP rs1870547 genotype (AG: *n* = 11; GG: *n* = 51) and *NLK* mRNA expression in neutrophils and PBMCs, respectively. **f** Sex-specific difference in the relation between rs4240847 genotype and *MAPKAPK2* mRNA expression in neutrophils. **g** The relation between *MAPKAPK2* SNP rs4240847 genotype and the type I response of neutrophils (CA: *n* = 15; AA: *n* = 47). Data are shown as means ± SEM. *P < 0.05; **P < 0.05. *P*-values were calculated by the Mann-Whitney U test. In all instances, *n* refers to the number of each group.

The activation of *IRF3*, *IRF7*, and *PRDM1* in IFN-α-responsive neutrophils was predicted to drive IFN-α-responsive element genes. Thus, we next explored the contribution of genetic factors to the IFN-α-responsive neutrophils under BU pathogenesis. Sex-specific stratification analysis was performed using Genome-wide association study (GWAS) data that we reported recently^59^. We first identified 572 and 1698 single nucleotide polymorphisms (SNPs) that were associated with female and male BU, respectively (*P* < 10^–5^) (Fig. 7a, b). After mapping these SNPs to the genes, male BU-specific SNPs were identified to be located in 120 genes (Fig. 7c). Among these genes that were specific for male BU patients, *STAT4*, *MAPKAPK2*, *HLA-E*, *NLK*, *PSMB9*, and *EHMT2* were associated with type I interferon pathway^60–64^. The female BU-specific SNPs were located in the 17 genes, such as genes encoding regulators of T cell differentiation (*BTNL2* and *ZBTB32*), HLA (*HLA-G*), and TF (*FOXO6*). We then assessed the expression of these genes in neutrophils from BU patients and HCs, respectively, using reverse transcription polymerase chain reaction (RT-PCR). In our study, the *MAPKAPK2* expression in male BU patients by neutrophils was significantly decreased and the *NLK* expression was markedly increased as compared with male HCs (Fig. 7d). However, no significantly different expression levels of these two genes were observed in females BU patients, as compared with female HCs (Fig. 7d). We interrogated if there was any potential immune-regulatory function of these male BU-specific susceptible SNPs in *MAPKAPK2* and *NLK* expression, and if the immune-regulatory function was specific to neutrophils. Thus, functional prediction on these SNPs was performed by using the 3DSNP database (Fig. 7c), and two SNPs (rs4240847 and rs1870547) were identified to be located in the enhancer of their locus in neutrophils as well as other cell types. In order to evaluate the functional significance of these two SNPs, we also compared the expression of *MAPKAPK2* and *NLK* in neutrophils and peripheral blood mononuclear cells (PBMCs) derived from 62 HCs with known genotypes of rs4240847 and rs1870547. Male BU-risk allele C of rs4240847 was associated with decreased mRNA expression of *MAPKAPK2* in neutrophils (Fig. 7e), but not in PBMCs, while rs1870547 failed to show any evidence of regulating *NLK* expression (Fig. 7e). Further gender stratification analysis revealed the specific association between risk allele C of rs4240847 with decreased mRNA expression of *MAPKAPK2* in males (Fig. 7f). In addition, the risk allele C of rs4240847 was associated with the diminished type I IFN responsive gene (*IFIT1*, *IFIT3*, and *IFI44L*) expression by neutrophils after treatment of IFN-α2a (Fig. 7g). These results suggested that male BU-specific SNPs were linked to the impairment on type I IFN signaling and response to type I IFN in neutrophils, probably resulting in the deficiency of IFN-α-responsive neutrophils.

### Male-specific circulating exosomes impair unconventional neutrophil subsets in BU

**Fig. 8:**
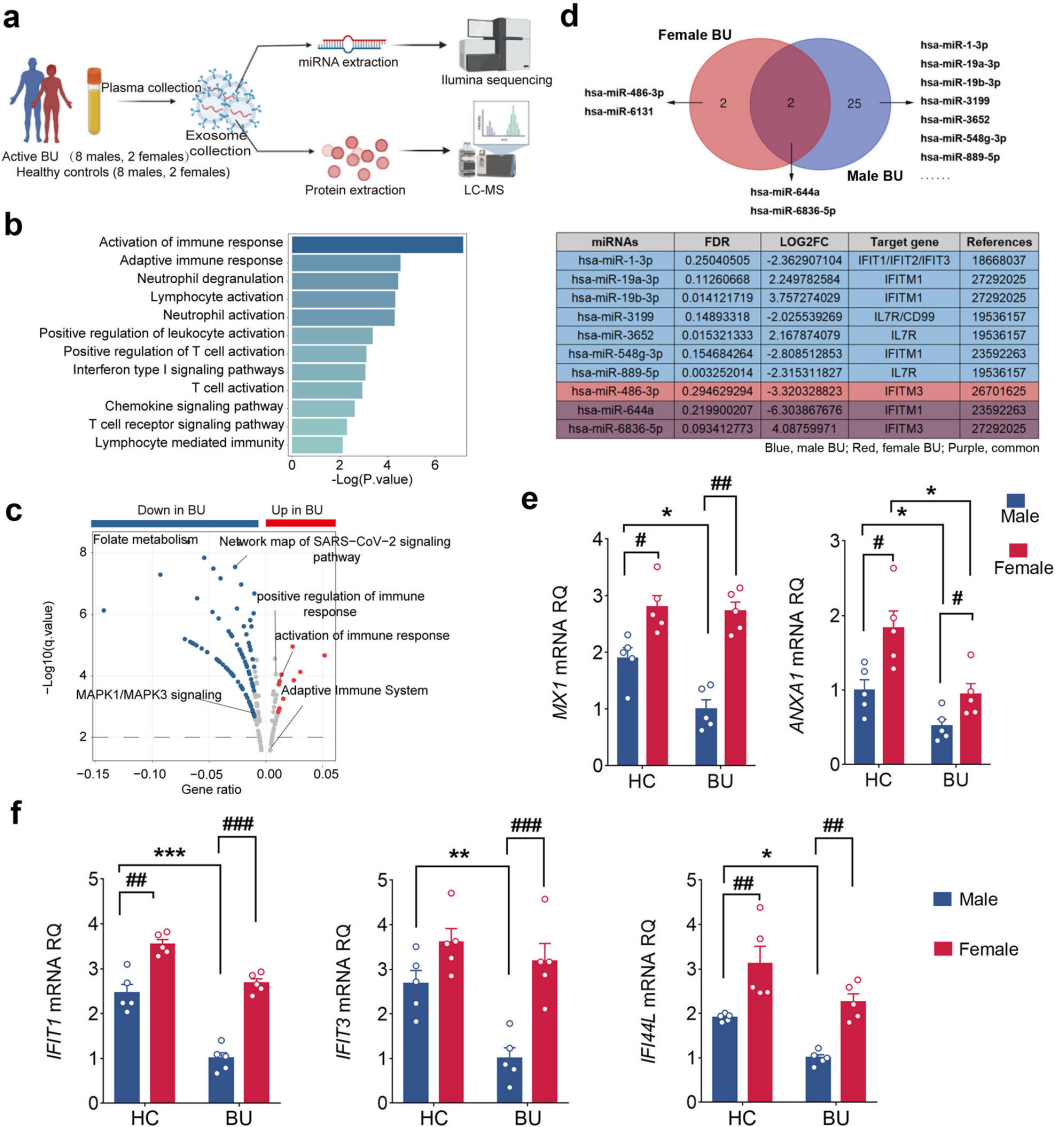
Male-specific circulating exosomes impair unconventional neutrophil subsets in BU. **a** Schematic of the experimental design for exosome study (female BU *n* = 2; male BU *n* = 8; female HCs *n* = 2; male HCs *n* = 8). **b** GO analysis of differentially expressed proteins between male patients and male HCs. **c** GO analysis of differentially expressed secretory proteins between male patients and male HCs. **d** Upper panel: specific and shared differentially expressed miRNAs (FDR < 0.3 and |LOG2FC | > 2) between male and female BU patients; lower panel: exosomal miRNAs specific for male patients and their target genes associated with type I interferon signaling pathway (*IFIT1*, *IFIT2*, *IFIT3*, and *IFITM1*) and regulation of T cell activation (*IL7R* and *CD99*). **e** Effects of exosomes from healthy individuals and active BU patients on the expression of *MX1* and *ANXA1* in neutrophils. Data are shown as means ± SEM. Data were analyzed by the Wilcoxon matched pairs test. *n* = 5 per group. **f** Effects of exosomes from healthy individuals and active BU patients on the type I interferon response of neutrophils. *n* = 5 per group. Data are shown as mean ± SEM. *BU vs HC: *P < 0.05; **P < 0.01, ***P < 0.001. ^#^Females vs males; ^#^*P* < 0.05; ^##^*P* < 0.01; ^###^*P* < 0.001 (**e**, **f**). Data were analyzed by the one-way ANOVA (Tukey’s multiple comparison test). In all instances, *n* refers to the number of each group.

Emerging evidence demonstrated the prominent contribution of dysregulated immune-environment to the functional heterogeneity of circulating neutrophils^20^. Thus, we assessed whether circulating factors influenced unconventional neutrophil subsets in male BU patients. The profiles of proteins and miRNAs in exosomes were analyzed, which were derived from the plasma of BU patients (2 females and 8 males) and HCs (2 females and 8 males) (Fig. 8a). Due to the scarce population of female BU patients, the differentially expressed exosomal proteins were only compared between male BU patients and male HCs, and the significant differences of proteins were identified involved in neutrophil activation and type I interferon pathway (Fig. 8b). We screened secretory proteins out of these different proteins according to the Human Protein Atlas, and found that the up-regulated secretory proteins in male BU patients were associated with positive regulation of immune response and neutrophil activation (Fig. 8c). Moreover, miRNA profiles collected from female and male patients were also analyzed, respectively. Twenty-seven differentially expressed miRNAs (FDR < 0.3 and |LOG2FC | > 2) were detected in male patients, whereas only 4 were identified in female patients (Fig. 8d). Unexpectedly, 25 out of 27 miRNAs were identified as male BU-specific and their target genes were associated with type I interferon signaling pathway (*IFIT1*, *IFIT2*, *IFIT3*, and *IFITM1*) and regulation of T cell activation (*IL7R* and *CD99*) (Fig. 8d), indicating the suppressive effects of exosomal miRNAs from male patients on IFN-α responding signaling and T cell response regulation signaling of neutrophils.

In order to further confirm the sex-specific effects of exosomes derived from BU patients on neutrophil subsets, we isolated exosomes from the plasma of BU patients (females and males) or healthy donors (females and males) first, and then cocultured with neutrophils. RT-PCR analysis of *MX1* and *ANXA1* mRNA expression in neutrophils was performed. We observed that the mRNA expression of *MX1* and *ANXA1* was markedly decreased in male BU patient-derived exosome group compared with male HCs-derived exosome group (Fig. 8e). However, in females, the difference of *MX1* expression between patients-derived exosome group and HCs-derived exosome group was not detected, although the expression of *ANXA1* was downregulated (Fig. 8e). We then interrogated whether collected exosomes could affect the type I IFN responses of neutrophils. As expected, exosomes derived from BU patients, especially male BU patients, could significantly downregulate the type I IFN responsive gene (*IFIT1*, *IFIT3*, and *IFI44L*) expression by neutrophils (Fig. 8f). Taken together, these data revealed the unfavorable circulating immune-microenvironment of male BU patients for unconventional neutrophils development, which further explained the markedly attenuated, even complete loss of unconventional neutrophils, that we observed in male BU patients.

## Discussion

We reported here 17,270 single-cell, genome-wide quantitative transcriptomes of human peripheral neutrophils in HCs and BU patients. We molecularly defined neutrophil subset phenotypes and their sex-specific differences, and concentrated on elucidating the functionalities of two unconventional neutrophil subsets. We further explored the mechanisms by which they drove inflammation in both genders of BU patients. We also revealed the male-specific vulnerability of neutrophil subsets and functional SNPs identified by GWAS and circulating hostile immune-microenvironment associated with BU pathogenesis.

Dysregulation in the phenotype and function of neutrophils has been recognized as key drivers in the onset and progression of BU pathogenesis^65^. Our work uncovered a stable unconventional neutrophil subset exhibiting a signature of type I Interferon responding pathway that was sex-specifically influenced during this disease. Another unconventional neutrophil subset was Annexin A1^+^ population and displayed an indispensable role in maintaining Th17–Treg cell balance and immune homeostasis. We further identified the sex-specific differences in neutrophil heterogeneity under auto-inflammatory condition. More importantly, we noticed that Annexin A1^+^ subset was hardly noted in male BU patients. Interestingly, immunomodulation with IFN-α2a, one of the members of type I interferon family known to improve morbidity in severe refractory BU patients^66^, was associated with global alteration of unconventional neutrophils as well as the enhancement of neutrophilic response to IFN-α and Annexin A1 signalings. A preferential expansion of unconventional neutrophil subsets was observed in females. These altered neutrophil subsets suggested a second route of the immunoregulatory effect of IFN-α2a on both neutrophils and systemic innate immune response. Thus, early initiation of IFN-α2a therapy is suggested to mitigate BU severity in both females and males^67^, however, for treating severe and relapsing BU, it has specifically therapeutic effects in female patients.

Multifactorial etiological factors, from both genetic and environmental origins, contribute to BU development^68,69^. A higher incidence of Behçet’s disease is found in the developing countries along the old Silk Route probably attributed to their relatively lower living and medical conditions which may result in abnormal neutrophil phenotype susceptibility to this disease^70^. Our work also showed both male BU-specific SNPs by GWAS stratification analysis, and male BU-specific hostile immune-microenvironment contributed to unconventional neutrophil subset dysregulation. Although rs4240847 is identified as an intron variant, it may impact the mRNA expression of *MAPKAPK2* via affecting the bindings of TF to the enhancer^71^. Further studies are needed to investigate the exact mechansims underlying the negative effects of both male patient-specific genetic factors and circulating exosomes on unconventional neutrophil subsets. These results provided additional evidence that male-specific complex factors constrained unconventional neutrophil expansion during BU pathogenesis, consequently leading to a higher incidence of certain lesions, severe disease activity and a poor visual outcome. Conversely, expanded unconventional neutrophils were associated with a relatively mild disease in females.

Several exciting avenues of this study remain, including investigating where and how neutrophil phenotype alteration occurs in response to IFN-α2a and during BU pathogenesis. As pre-neutrophils in the bone marrow become non-mitotic and can enter the peripheral blood in an early or immature form with banded morphology^72^, we speculate that neutrophil subset alterations occur after they enter circulation; however, this needs formal testing. Besides unconventional subsets, highlighted in this study, our scRNA-seq analysis showed the sex-specific differences in three conventional subsets under diseased and healthy conditions, however, their exact functionalities and contributions to the pathogenesis of auto-inflammatory diseases are still not completely understood and needed to be addressed in the future study.

Our study field now has a near-complete census of circulating and tissue-infiltrating neutrophils. Our work markedly expands sex-specific and disease-specific heterogeneity and functional subsets of human neutrophils, and also facilitates translational opportunities. Defining mechanisms of neutrophil subset alteration sex-specifically and disease-specifically will enable researchers to therapeutically target unwanted neutrophil subsets or enhance beneficial neutrophil subsets in the treatment of relevant diseases. Furthermore, we are hopeful that our investigation will inform the development of the generation of disease-specific and sex-specific beneficial neutrophils from bone marrow precursors in vitro, with the ultimate aim of reinfusing them into individuals with refractory auto-inflammatory BU, as an autologous cellular therapy.

## Materials and methods

### Study design

The aim of this study was to define sex-specific heterogeneity of human circulating neutrophils, and to further explore the underlying mechanism by which they drive sex-specific incidence and outcomes of auto-inflammatory BU. The first objective was illustrated by scRNA-seq performed on circulating neutrophils from healthy individuals and auto-inflammatory BU patients, describing the sex-specific heterogeneity in neutrophil composition that was associated with immunological outcome of BU. More specifically, unconventional neutrophil subsets were identified as a major determinant of male-biased vulnerability to BU, and biologic IFN-α2a could directly promote unconventional neutrophil subsets to a more immune-regulatory phenotype for the treatment of auto-inflammatory BU. The second objective was investigated by GWAS analysis and proteomics/miRNA analysis of circulating exosomes, determining the negative effects of both genetic factors and circulating exosomes on unconventional neutrophil subsets, which also contributed to male-biased vulnerability to disease. These findings shed new light on the cellular processes of neutrophils contributing to sex-specifically immunological outcome of BU, which might guide the development of rationally sex-specific immunotherapies targeting on neutrophils to benefit patients with auto-inflammatory diseases.

### Subject details

For this study, the diagnosis of BU was based on diagnostic criteria designed by the ICBD^43^. The diagnosis of VKH disease was carried out according to the revised criteria of an international nomenclature committee and modified criteria developed by our team^44,45^. Active BU was defined as active intraocular inflammation in association with at least one of active systemic manifestations such as oral ulceration, genital ulceration, and skin lesion. Active VKH disease was defined as active inflammation in the anterior segment, posterior segment or both^73^. Patients involved in this study had stopped taking immunosuppressive medicines for at least three weeks or only used less than 20 mg/d prednisone, prior to blood sampling when they visited our clinic. For control subjects, HCs with systemic diseases and other immune-mediated diseases, as well as those on medication within the past month, were excluded. Detailed data, including age, gender, and clinical manifestations, were obtained from all patients and HCs.

### Ethics statements

The whole process of this study was approved by the Ethics Committee of the First Affiliated Hospital of Chongqing Medical University (permit number 2018-048). Consent was obtained from all patients and HCs.

### Neutrophil isolation

Whole blood from BU patients and HCs was collected in heparin tubes. Within 3 h after sample collection, we first diluted 4 mL blood with the same amount of phosphate buffer saline (PBS) solution, then carefully layered it over Ficoll (TBD) in a 15 mL tube, and centrifuged it with Ficoll at 350× *g* for 20 min at 4 °C. The neutrophil layer was collected by quickly pouring the contents into a new 15 mL tube. After neutrophil collection, red blood cell (RBC) lysis (Beyotime) was used to obtain pure neutrophils. The cell suspension was washed twice with PBS and stored for subsequent processing.

### ScRNA-seq analysis

Neutrophils were collected from 18 patients with active BU (8 females, 10 males) and 16 HCs (8 females, 8 males) (Supplementary Table S2). Neutrophils from 2 to 4 same-donors were pooled (3 female BU pools, 3 male BU pools, 4 female HC pools, and 3 male HC pools). Before processing them for scRNA-seq, the purity and viability of the neutrophils from each pooled samples (> 95% purity, > 90% viability, respectively) was determined by flow cytometry (CD15^+^CD11b^+^) and trypan blue exclusion test, respectively. Approximately 10,000 single cells from each pooled samples were sequenced using the 10x Genomics platform (10x Genomics). The 10x Genomics 3′ mRNA single-cell method was used. Published data of scRNA-seq on mice-derived neutrophils, used in this study, were retrieved from GEO: GSE137540. Cells were encapsulated using microfluidic technology and barcoded using a unique molecular identifier (UMI). We prepared the cDNA and performed the sequencing analysis using the HiSeq 2000 sequencing system (Illumina), according to the manufacturer’s directions. Quality control of the raw reads was assessed by FASTQC software (http://www.bioinformatics.babraham.ac.uk/projects/fastqc/). Data were demultiplexed using Cell Ranger software (version 2.2.0; https://support.10xgenomics.com/single-cell-gene-expression/software/downloads/latest), which generated FAST-Q files that were aligned to a human reference genome (GrCh38) using STAR^74^. UMI counts were summarized using Ensembl gene annotation GTF file obtained using the UCSC Table Browser tool^75^. We employed the R package Seurat (version 4.0.2) for the data analysis of scRNA-seq. After normalization and quality control of the data, UMAP was used for dimensional reduction and clustering, using ten principal components as input and employing the graph-based shared nearest neighbor (SNN) method. The clustering results for each sample were visualized using UMAP.

### ScRNA-seq cluster marker detection and differential expression analysis

To determine cluster marker genes, cells from each cell cluster were compared against all other cells in the experiment. The wrapper function of “FindAllMarkers” in Seurat was used for statistical testing, with default parameters for filtering out genes below a minimum log-fold change threshold of 0.25 and infrequently (10% of cells) expressed genes. Genes with adjusted *P*-values lower than 0.05 were considered as marker genes. The results were presented as dot plots and heatmaps using the R packages Seurat (https://github.com/satijalab/seurat) and ggplot2 (https://github.com/tidyverse/ggplot2), respectively. All the differential expression analyses under different conditions were performed by the function of “FindMarkers” in Seurat. Wilcoxon rank-sum test was used to estimate adjusted *P* values, and genes with adjusted *P*-values lower than 0.05 were considered significantly differentially expressed.

### GO and pathway enrichment analyses

For all cases, enrichment analysis on DEGs or cluster markers was performed by Metascape^76^. The GO terms and pathways with adjusted *P*-values lower than 0.05 were considered statistically significant. To identify the specific biological pathways enriched in each cell cluster, GSEA analysis was performed using the msigdbr and fgsea packages (https://www.gsea-msigdb.org/gsea/msigdb/index.jsp).

### Pseudotime trajectory analysis

Pseudotime trajectory analysis was performed with the R package monocle2 (v2.8.0; http://cole-trapnell-lab.github.io/monocle-release/)^77^. Neutrophils from BU patients and HCs were clustered as described in “ScRNA-seq analysis”. The cell trajectory was then captured using the “orderCells” function, with the starting pseudotime state denoted as the end of the trajectory that was found to be enriched for neutrophil clusters. Detection of the genes that significantly covaried with pseudotime was based on a log-likelihood ratio test between the model formula that included cell pseudotime and a reduced model formula. Additional model covariates were included in the residual model formula according to the steps in “ScRNA-seq analysis”. Benjamini–Hochberg multiple testing correction was used to calculate FDR, and genes with FDR < 5% were considered to vary significantly with pseudotime.

### Inferring cell–cell communication

Inter-cellular and internal signalings among different clusters were inferred by CellCall^78^. This is a toolkit that analyzes inter-cellular communication networks and internal regulatory signals by combining the expression of ligands/receptors for certain ligand–receptor (L–R) pairs. We used a pathway-activity analysis method to explore the main pathways involved in communication between clusters. Genes that were expressed in less than 10% of the cells of a certain type were excluded from this study.

### Expression patterns of TFs in human neutrophils

The normalized single-cell gene expression matrix was initially filtered to exclude all genes detected in fewer than 3% of cell numbers. The RcisTarget database, which contains TF motif scores for gene promoters and approximate transcription start sites for the hg38 human reference genome, was downloaded from https://resources.aertslab.org/cistarget/databases/homo_sapiens/hg38/refseq_r80/mc9nr/gene_based/, and the expression matrix was further filtered to include only genes available in the RcisTarget database. The remaining genes were used to compose a gene–gene correlation matrix for co-expression module detection using the random forest-based GENIE3 algorithm. Besides, the R package SCENIC^79^ was used to perform TF network analysis and detect co-expression modules enriched for the target genes of each candidate TF from the RcisTarget database. Enrichment analysis for different TF target genes was performed using Metascape. The GO terms with adjusted *P* values lower than 0.05 were considered statistically significant.

### Retrospective study design

For this study, we retrospectively analyzed the data of a total number of 1881 BU patients (310 females, 1571 males), including both rural and urban residents, from the uveitis department of the First Affiliated Hospital of Chongqing Medical University. Detailed data, including age, gender, ocular manifestations, systemic manifestations, BCVA, and treatment at presentation, were obtained from all patients.

#### Visual prognosis

Of these patients, 1409 (226 females, 1183 males) received follow-up examinations more than one month after treatment and were eligible for the risk-evaluation study. Besides, we performed Kaplan-Meier survival analysis to assess the risks of poor vision (0.05 ≤ BCVA of the better eye < 0.3) and blindness (BCVA of the better eye < 0.05) in all of these patients. All participants provided informed consent to participate in this study. The results were analyzed using SPSS Statistics (version 23; SPSS, IBM).

#### Ocular manifestation assessment

Among these patients, 1615 (257 females, 1358 males) received detailed assessment of both FFA and OCT when they visited our clinic. We compared the incidence rates of retinal vasculitis, papillitis, macular edema, retinal atrophy and optic atrophy between female and male patients using the χ^2^ test in SPSS Statistics.

#### Systemic manifestation assessment

Systemic manifestations were assessed in all BU patients, including oral ulceration, genital ulceration, skin lesions, arthritis, CNS involvement, gastrointestinal ulceration, perineal abscess, and thrombophlebitis. Then we compared the incidence rates of these systemic manifestations between female and male patients using the χ^2^ test in SPSS Statistics.

#### Routine blood tests

To avoid pharmacological perturbation in this comparison, we included 78 active BU patients (14 females, 64 males) and 29 inactive BU patients (10 females, 19 males) who did not receive medications at least for 2 weeks before sampling. We performed routine blood tests for these patients, including counts of neutrophils, lymphocytes, monocytes, eosinophils and basophils, to evaluate the relationship between these parameters and BU. For this evaluation, we chose 74 patients with active VKH disease (36 females, 38 males) and 78 HCs (14 females, 64 males) as control subjects. We non-specifically chose HCs from Health Examination Center of the First Affiliated Hospital of Chongqing Medical University to retrospectively analyze their routine blood test data.

### Neutrophil apoptosis assay

Using the freshly isolated neutrophils, we performed staining to detect neutrophil surface markers and apoptotic cells. The antibody clones (Supplementary Table S1) used for flow cytometric analysis were: Annexin V-APC (Vazyme), propidium iodide (PI; Vazyme), anti-human CD11b-PE (Biolegend), and anti-human CD15-PE-Cy7 (Biolegend). The mixture of these staining antibodies was directly added to suspensions containing 5 × 10^5^ freshly isolated neutrophils and kept for 20 min at room temperature. After this incubation, we washed the cells with PBS and prepared them for analysis.

### PBMC and CD4^+^ T cell isolation

PBMCs were isolated from the heparinized whole blood that was collected from donors according to the process in “Neutrophil isolation”. To isolate PBMCs, whole blood was diluted at a ratio of 1∶1 in PBS and then layered over Ficoll (TBD). The samples were centrifuged at 300× *g* for 20 min at room temperature. After extraction, the PBMCs were washed twice with PBS and then centrifuged at 2000 rpm for 10 min. The CD4^+^ T cells were separated from PBMCs using positive selection MACS-kits, according to the manufacturer’s instructions (Miltenyi Biotec).

### Enzyme-linked immunosorbent assay (ELISA)

Neutrophils were isolated from the whole blood of donors, as described above in “Neutrophil isolation”. We plated 5 × 10^5^ isolated neutrophils in 500 µL culture medium consisting of RPMI 1640 medium, 100 U/mL penicillin/streptomycin, and 10% fetal bovine serum in each well of a 48-well plate. The supernatant of the cells was collected after stimulation with 100 nM phorbol 12-myristate 13-acetate (PMA; Sigma) for 4 h at 37 °C. Cell-free supernatants were collected for MPO secretion analysis by ELISA. Duoset ELISA development kits (R&D Systems) were used to quantify MPO secretion in the supernatant, according to the manufacturer’s guidelines. NE produced by neutrophils was detected by NETosis Assay (Abcam), according to the manufacturer’s guidelines.

CD4^+^ T cells were isolated from the whole blood of donors, as described above in “PBMC and CD4^+^ T cell isolation”. We plated 1 × 10^6^ isolated CD4^+^ T cells in 500 µL culture medium consisting of RPMI 1640 medium, 100 U/mL penicillin/streptomycin, and 10% fetal bovine serum in each well of a 48-well plate. The supernatant of cells was collected after stimulation with anti-CD3/CD28 after 3 days. ELISA kits (Elabscience) were used to quantify IL-10, IFN-γ, and IL-17 secretion in the supernatant, according to the manufacturer’s guidelines.

### T cell proliferation assay

For T cell proliferation by CSFE staining, CD4^+^ T cells were incubated with anti-CD3/CD28 stimulation for 24 h. After stimulation, CD4^+^ T cells were labeled with 10 μM of CFSE (MCE) for 15 min. Before analysis, the cells were washed and resuspended in 200 µL PBS.

### Th1/Th17 and Treg cell detection in CD4^+^ T cells using flow cytometry

The CD4^+^ T cells were isolated from the whole blood of donors in the “PBMC and CD4^+^ T cell isolation” stage. After isolation, staining was performed in three separate panels for Th1, Th17 and Treg cells. The exact antibody clones (Supplementary Table S1) used for flow cytometric analysis were as follows: anti-human IFN-γ-PE-Cy7 for Th1 cell detection, anti-human IL-17A-PE for Th17 cell detection, plus anti-human FOXP3-PE for Treg cell detection. All antibodies were purchased from Biolegend. For the Th1 and Th17 cells, we seeded 5 × 10^5^ freshly isolated CD4^+^ T cells in 500 µL culture medium containing RPMI 1640 medium, 100 U/mL penicillin/streptomycin, and 10% fetal bovine serum with 1 µL cell activation cocktail with brefeldin A (Biolegend) for 6 h at 37 °C. Subsequently, the cells were washed with permeabilization buffer (Invitrogen) and blocked with fixation buffer (Invitrogen) for 40 min. Then the IFN-γ and IL-17A antibodies were added to the cell suspension and held for 30 min at 4 °C. Before analysis, the cells were washed and resuspended in 200 µL permeabilization buffer and stored at 4 °C overnight. Regarding Treg cells, after washing, the cells were resuspended in a mixture containing fixation/permeabilization concentrate buffer (Invitrogen) and fixation/permeabilization diluent (Invitrogen). Finally, FOXP3 antibody was added into the cell suspension and then kept at 4 °C for 1 h. After incubation, the cells were washed and resuspended in 200 µL permeabilization buffer at 4 °C in preparation for flow cytometric analysis.

### Immunofluorescence staining for detection of NETs in neutrophils

After stimulation with PMA, slides with neutrophils were washed by PBS and fixed with 4% paraformaldehyde in PBS. Blocking was performed with 10% normal goat serum. NETs were detected with mouse anti-MPO (Abcam) in 10% normal goat serum. Slides were incubated with goat anti-mouse IgG (DyLight 488, ImmunoWay) in PBS. Images were obtained with a confocal microscope (MOLECULAR DEVICES).

### RT-PCR

Total RNA was isolated from neutrophils using TRIzol reagent (Invitrogen). The PrimeScript RT kit (Takara Biotechnology) was used to reverse the extracted RNA into cDNA. We used the ABI Prism 7500 system with SYBR Premix (Bio-Rad) to detect and analyze gene expression. The relative expression of target genes was quantified using the 2^*−*^^ΔΔCt^ method with β-actin acting as the internal reference. Supplementary Table S31 presents a list of the primers used.

### CD4^+^ T cell co-culture experiment

We extracted CD4^+^ T cells from PBMCs in the process described above. For co-culture experiments, 5 × 10^5^ CD4^+^ T cells were seeded into each well of a 48-well plate and co-cultured with 500 µL IFN-α2a (10 ng/mL) preincubated neutrophil culture supernatant for 24 h before flow cytometry.

### Mice

The animal studies were approved by the Committee of Animal Care of Third Military Medical University (Army Medical University) and the Ethics Committee of the First Affiliated Hospital of Chongqing Medical University. C57BL/6 J mice were obtained from Laboratory Animal Center of Third Military Medical University. *Ly6g*^Cre-ERT2^ (*Ly6g*^Cre/+^) and *Anxa1*^em1cyagen^ (*Anxa1*^fl/fl^) mice (5-week-old, on C57BL/6 J background) were purchased from Cyagen Biosciences. *Ly6g*^Cre/+^-*Anxa1*^fl/fl^ mice, the model for neutrophil-specific deletion of *Anxa1*, were generated by crossing-breeding of *Ly6g*^Cre/+^ mice with *Anxa1*^fl/fl^ mice. To induce the conditional *Anxa1* ablation on neutrophils, *Ly6g*^Cre/+^-*Anxa1*^fl/fl^ mice were injected intraperitoneally with tamoxifen (20 mg/500 µL; Sigma-Aldrich) in corn oil (Sigma-Aldrich) for 7 consecutive days^80^. After a wash out period of at least 7 days, the genotype of CD11b^+^Ly6g^+^ neutrophils, collected by FACS, were confirmed by PCR (primers provided in the Supplementary Table S31) and western blotting assay (anti-Annexin A1, Abcam). Mice with successful Cre-driven deletion (*Anxa1*^ΔLy6g^) were applied in further experiments, and *Anxa1*-floxed homozygous and Cre-negative littermates (*Anxa1*^fl/fl^) were used as control. All mice were specific pathogen free (SPF) grade, maintained under the standard ambient temperature (20–26 °C) and humidity (50–60%) with strict 12 h light/12 h dark cycle. Inhalation anesthetization was performed with 3% isoflurane (Merck) mixed in oxygen for experiments in vivo. The mutant mice and their littermate controls used in this study were housed in separate cages.

### Immunofluorescence staining for Annexin A1^+^ neutrophils

Due to the low proportion of Annexin A1^+^ neutrophils in the pan neutrophils, Annexin A1^+^ neutrophils were first enriched using FACS. After enrichment, neutrophils were stained with anti-CD66b (Biolegend) and anti-Annexin A1-APC (AssayLite) as described above in “Immunofluorescence staining for detection of NETs in neutrophils”. Images were obtained with a confocal microscope (Nikon).

### EAU induction

Six-week-old female and male C57BL/6 J, *Anxa1*^fl/fl^ or *Anxa1*^ΔLy6g^ mice were immunized with 500 μg of IRBP651-670 (Sangon Biotech) emulsified in complete Freund’s adjuvant (CFA; Sigma-Aldrich) and supplemented with 5.0 mg/mL Mycobacterium tuberculosis (MTB) strains. Intraperitoneal injection of 500 ng of pertussis toxin (List Biological) was performed at 0 h and 24 h. Splenic cells were collected from the mice at 1, 2, 3, and 4 weeks after immunization. We used mice without EAU induction as controls. The mice were housed in a dedicated pathogen-free facility maintained at 24 ± 2 °C under a 12 h light/12 h dark cycle. For IFN-α2a treatment, mice were given intraperitoneal injections of IFN-α2a (2,000 IU, Peprotech) daily for 2 weeks starting from day 14 after immunization. The clinical and histopathological scores of EAU were evaluated as described in the previous study^81^. For adoptive transfer in murine model, freshly magnetically isolated and qualified neutrophils were resuspended in sterile PBS at a concentration of 1 × 10^4^ cells/µL, and 1 × 10^6^ cells were intravenously injected into the recipient mice through tail vein.

### Th1/Th17 and Treg cell detection in splenic lymphocytes using flow cytometry

The mouse spleens were crushed using a syringe through a 70 μm cell strainer. The single-cell suspension was first diluted in a 1∶1 ratio with PBS and then layered over Ficoll (TBD). After centrifugation at 350× *g* for 15 min, the lymphocyte layer was collected for the subsequent flow cytometric analysis. Similar to the panels used in human sample experiments, the antibody clones used for flow cytometric analysis were: anti-mouse CD4-APC, anti-mouse IFN-γ-PE-Cy7, anti-mouse IL-17-PE, and anti-mouse FOXP3-PE. All antibodies were purchased from Biolegend. After incubation with surface marker (CD4) antibodies, the cells were washed, fixed, permeabilized, and incubated with intra-cellular or intranuclear staining. These procedures were carried out in the same way as experiments with human samples.

### Quantification of Annexin A1 in splenic neutrophils using flow cytometry

The splenic cell suspension was collected in the manner described above. After centrifugation at 2000 rpm for 10 min, the cells were washed with RBC lysis and resuspended in PBS in preparation for flow cytometric analysis. A mixture of surface marker antibodies (Supplementary Table S1), comprising anti-mouse Ly6G-FITC (Biolegend), anti-mouse CD11b-PE (Biolegend), and anti-mouse Annexin A1-APC (AssayLite), was added to cell suspensions and held at room temperature for 20 min. Following washing, the cell samples were resuspended in 200 µL PBS and prepared for flow cytometric analysis.

### GWAS genotyping and analysis

We investigated susceptible SNP difference either between female BU patients and female controls or between male BU patients and male controls in the context of the GWAS data published recently^59^. The association of each SNP with BU in the GWAS was analyzed using an additive model in the logistic regression with PLINK (version 1.07). We adjusted the odds ratios (ORs) and 95% confidence intervals (CIs) for the top 10 eigenvectors in the logistic regression analysis. A corresponding SNP *P* value of < 1.0 × 10^−5^ was judged as suggestive evidence of association with disease. The specific disease-related genes for female BU and male BU were depicted using Venn diagrams (http://bioinformatics.psb.ugent.be/webtools/Venn/).

### DNA extraction and genotyping

Genomic DNA was extracted with a QIAamp DNA Blood Mini Kit (QIAGEN). Besides, we analyzed genotyping using the iPLEX Gold assay and designed the primers with MassARRAY Assay Design software (Sequenom). We also used TYPER software (version 4.0; Sequenom) to analyze the experimental data.

### Plasma exosome isolation and quantification

Plasma was collected after centrifugation at 2000× *g* for 10 min and then stored at −80 °C for later use. Exosomes were isolated from plasma samples with the Q3 Exosome Isolation Kit (Wayen Biotechnologies) according to the manufacturer’s guidelines. Nanoparticle tracking analysis (NTA) was used to detect the particle size and concentration of exosomes.

### Exosomal RNA isolation and sequencing

We investigated differentiallly expressed exosomal miRNAs either between female BU patients and female controls or between male BU patients and male controls based on our published exsomal miRNA sequencing data^82^. Exosomal RNA was isolated using the RNeasy Micro Kit (QIAGEN), according to the manufacturer’s directions. The libraries were prepared using the NEBNext Multiplex Small RNA Library Prep Set for Illumina (NEB), according to the instructions. Single-end 50-bp sequencing was performed on the Illumina HiSeq 2500 platform. Analysis of count data was performed using the DESeq2 package (version 1.0.19), and analyses were stratified by gender using edgeR. Differentially expressed miRNAs were identified based on criteria set as an absolute 2-fold change, and an FDR value < 1%. We identified the target genes of miRNAs by using five databases/algorithms: miRanda, miRDB, miRWalk, RNA22, and Targetscan.

### Exosome protein extraction and proteomic analysis

We investigated differentiallly expressed exosomal proteins either between female BU patients and female controls or between male BU patients and male controls based on our published exosomal proteomic study data^83^. The exosome pellets were lysed in a lysis buffer containing protease inhibitors (Thermo Fisher), followed by 1 min of sonication using an ultrasonic processor. The lysate was then centrifuged at 13,000 rpm for 20 min at 4 °C, and the supernatants were collected. The dried and desalted peptides of the exosomes were extracted for mass spectrometry analysis. The mass spectral (MS)/MS data was analyzed using MaxQuant software (version 1.5.8.3; Max-Planck Institute for Biochemistry). Proteins were identified using Swiss-Prot databases and quantified using label-free quantification. The mass tolerance value for the fragment ions was set to 0.05 Da. The FDR value was set to < 0.3, and the differential expression threshold to a 2-fold change.

### Treatment of neurophils with exosomes

Neutrophils (5 × 10^5^) were isolated from HC blood as described above in “Neutrophil isolation” and cultured in 500 µL exosome-free medium (Umibio, China). Exosome was isolated from the 2 mL plasma of patients or HC as described in “plasma exosome isolation” and resuspended in the PBS. After quantification by NTA, the exosome was added into each well at 2 × 10^10^ particles/mL.

### Statistical analysis

All data are expressed as means ± SEM. Data were analyzed and visualized using GraphPad Prism 6.0 (GraphPad software Inc., San Diego, CA, USA). Statistical significance and sample capacity (*n*) were detailed in corresponding figure legends.

### Supplementary information

Supplementary figures

Supplementary tables

## Data Availability

All raw scRNA-seq have been deposited with GSA under accession number: HRA002148. Processed data are available as Supplementary Tables. Published data of scRNA-seq on mouse-derived neutrophils, used in this study, were retrieved from GEO: GSE137540.
