# Supplementary material for: Sex-specific circulating unconventional neutrophils determine immunological outcome of auto-inflammatory Behçet’s uveitis
Source: Cell Discov. 2024 May 4;10:47. doi: 10.1038/s41421-024-00671-2 (PMC11069589; doi:10.1038/s41421-024-00671-2)
Supplement: Supplementary file 1 — Supplementary figures [file 41421_2024_671_MOESM1_ESM.pdf]

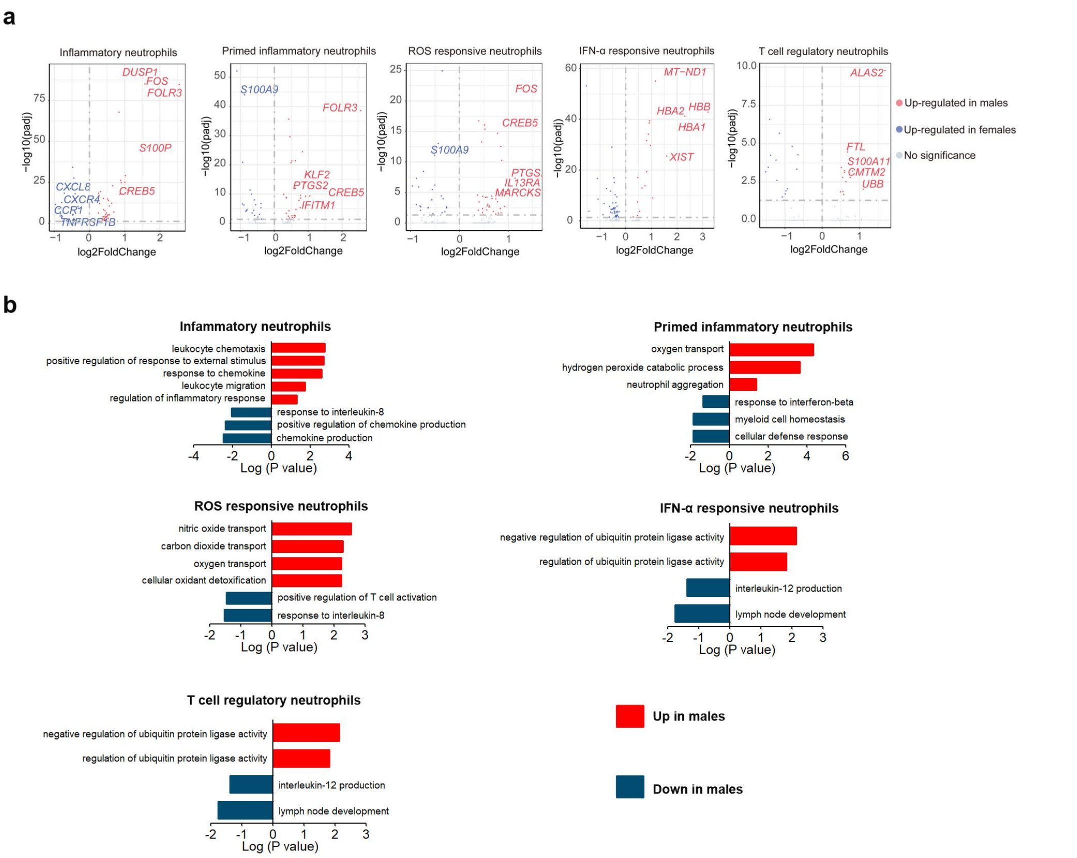

Fig S1. Sex-specific DEGs among neutrophil subsets.

a Volcano plot showing sex-specific DEGs within neutrophil subsets from healthy donors. **b** GO analysis of sex-specific DEGs within neutrophil subsets from healthy donors.

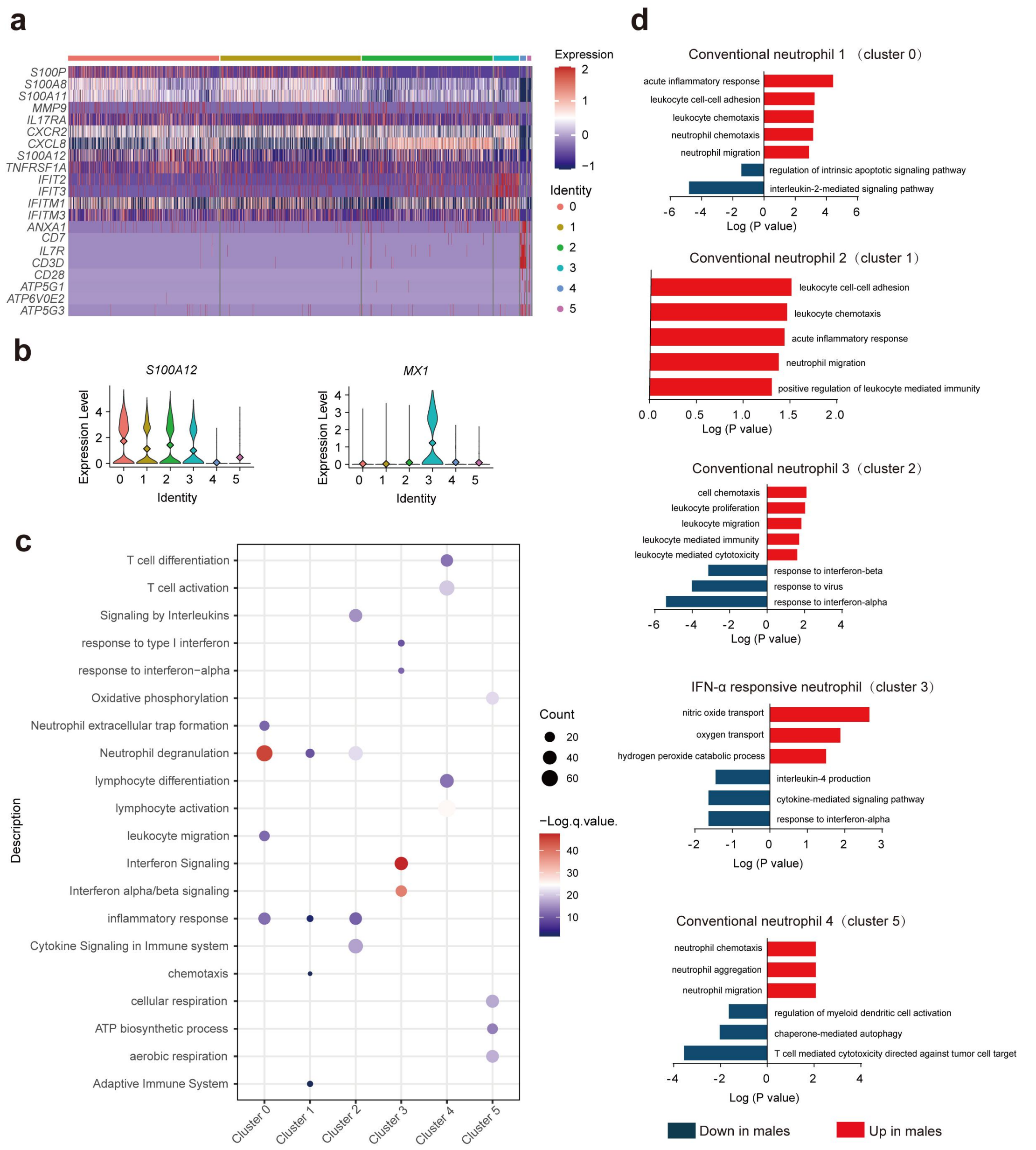

Fig S2. Gene signatures of neutrophil subsets in Behçet's uveitis patients.

a Heatmap showing expression of selected cluster marker genes in different neutrophil subsets from female (n = 8) and male BU patients (n = 10). **b** violin plot showing the specific expression of *S100A12* and *MX1* in the different neutrophil subsets. **c** Dotplot heatmap showing GO biological process terms enriched in cluster marker genes. Cluster 0: conventional neutrophils 1; Cluster 1: conventional neutrophils 2; Cluster 2: conventional neutrophils 3; Cluster 3: IFN-α responsive neutrophils; Cluster 4: T cell regulatory neutrophils; Cluster 5: conventional neutrophils 4. **d** GO analysis of sex-specific DEGs within neutrophil subsets from patients.

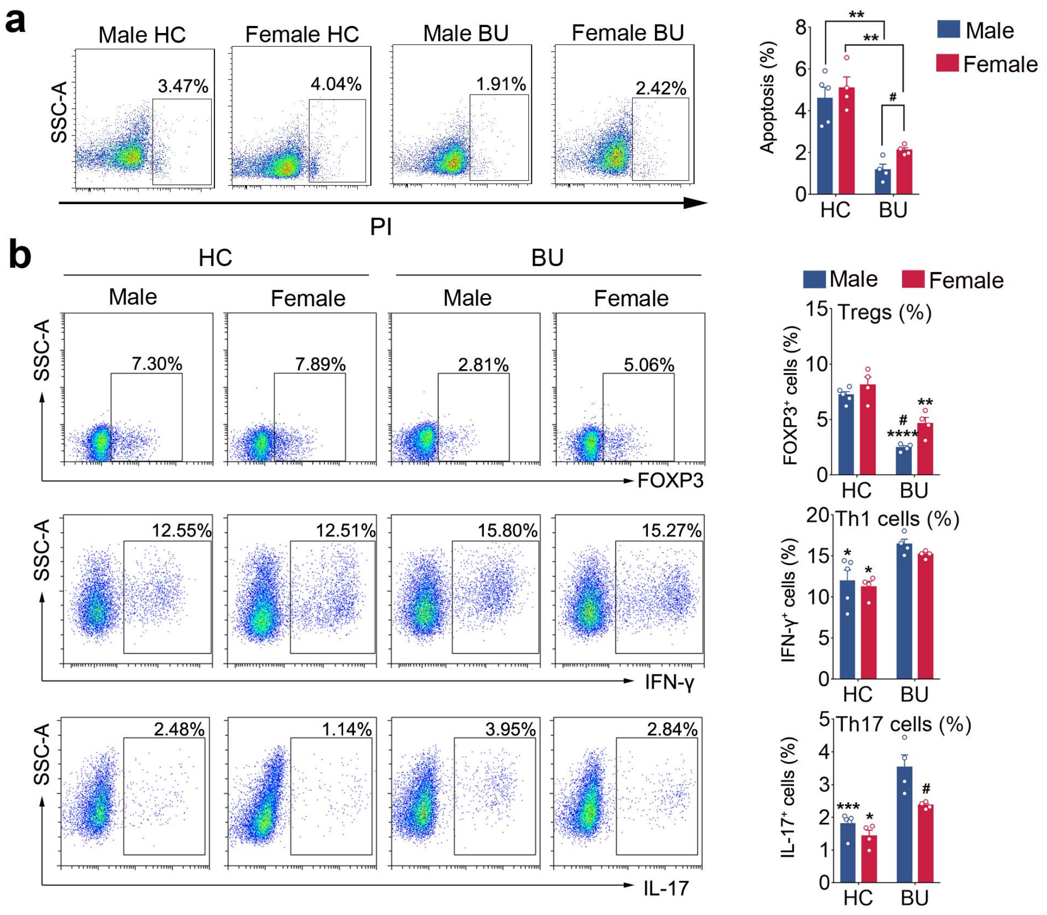

Fig S3. Aberrant circulating neutrophil and T cell responses in Behçet's uveitis patients. **a** Apoptosis status of peripheral neutrophils from active BU patients (female: n = 4; male: n = 4) and HCs (female: n = 4; male: n = 5). **b** The percentages of Th1/Th17 cells and Tregs from active BU patients (female: n = 4; male: n = 4) and HCs (female: n = 4; male: n = 5). Data are shown as mean ± SEM. *patients vs HCs: *$P < 0.05$; **$P < 0.01$; ***$P < 0.001$; ****$P < 0.0001$; #females vs males: #$P < 0.05$. P values were calculated by the one-way ANOVA (Tukey's multiple comparison test).

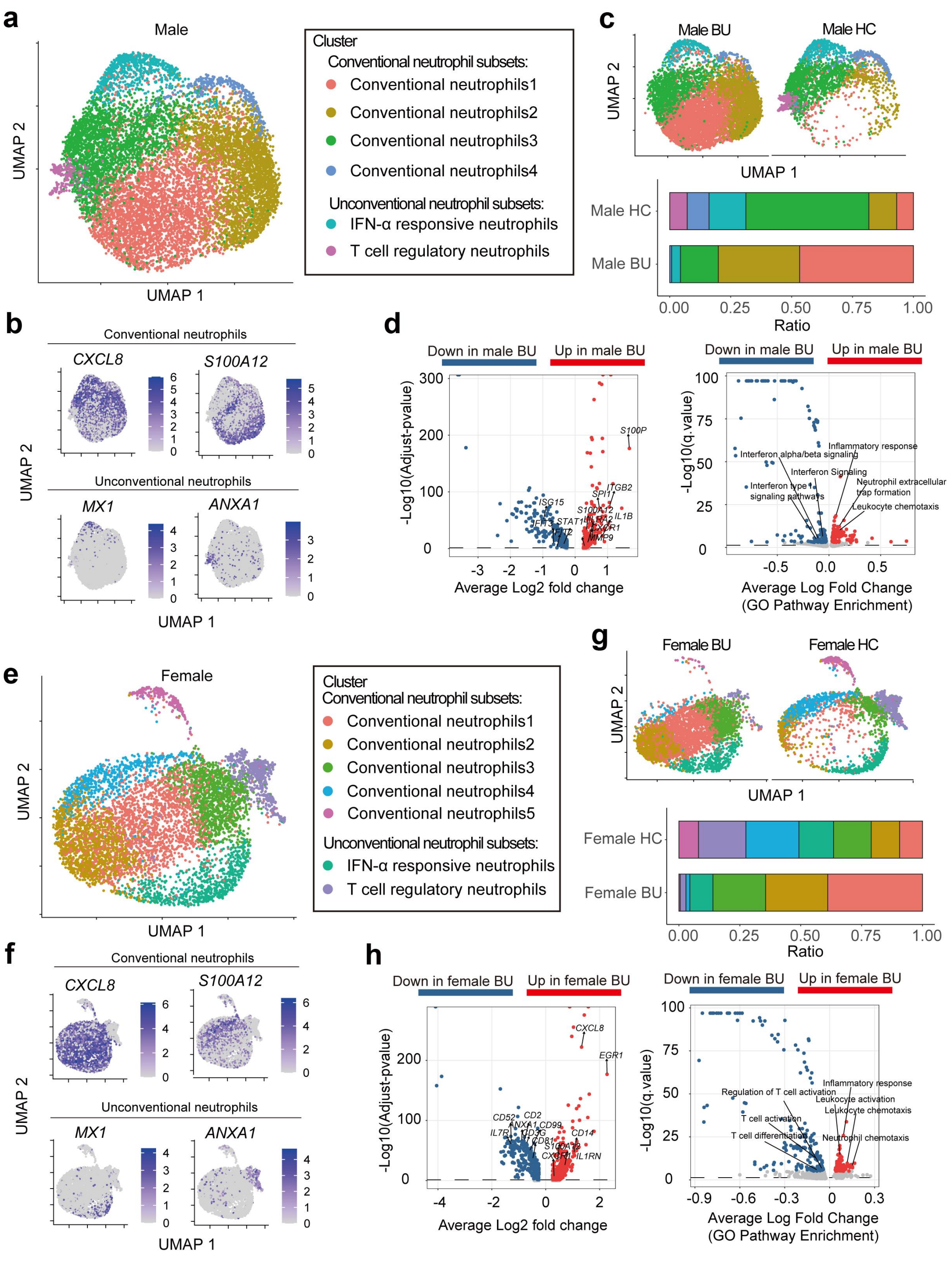

Fig S4. Comparative analysis of human neutrophils from males and females.

a UMAP visualization of neutrophils from male BU patients (*n* = 10) and male HCs (*n* = 8). **b** UMAP plot overlays showing selected gene expression distribution. **c** Upper panel: UMAP visualization of neutrophils from male HCs (*n* = 8) and male BU patients (*n* = 10), respectively; Lower panel: Bar plot of the proportions of different neutrophil subsets shown in male BU patients and male HCs, respectively. **d** Left panel: Volcano Plot showing the DEGs of neutrophils between male BU patients (*n* = 10) and male HCs (*n* = 8); Right panel: GO

analysis on the DEGs of neutrophils. **e** UMAP visualization of neutrophils from female BU patients (*n* = 8) and female HCs (*n* = 8). **f** UMAP plot overlays showing selected gene expression distribution across clusters. **g** Upper Panle: UMAP visualization of neutrophils from female HCs (*n* = 8) and female BU patients (*n* = 8), respectively; Lower panel: Bar plot of the proportions of different neutrophil subsets shown in female BU patients and female HCs, respectively. **h** Left panel: Volcano Plot showing the DEGs of neutrophils between female BU patients and female HCs; Right panel: GO analysis on the DEGs of neutrophils.

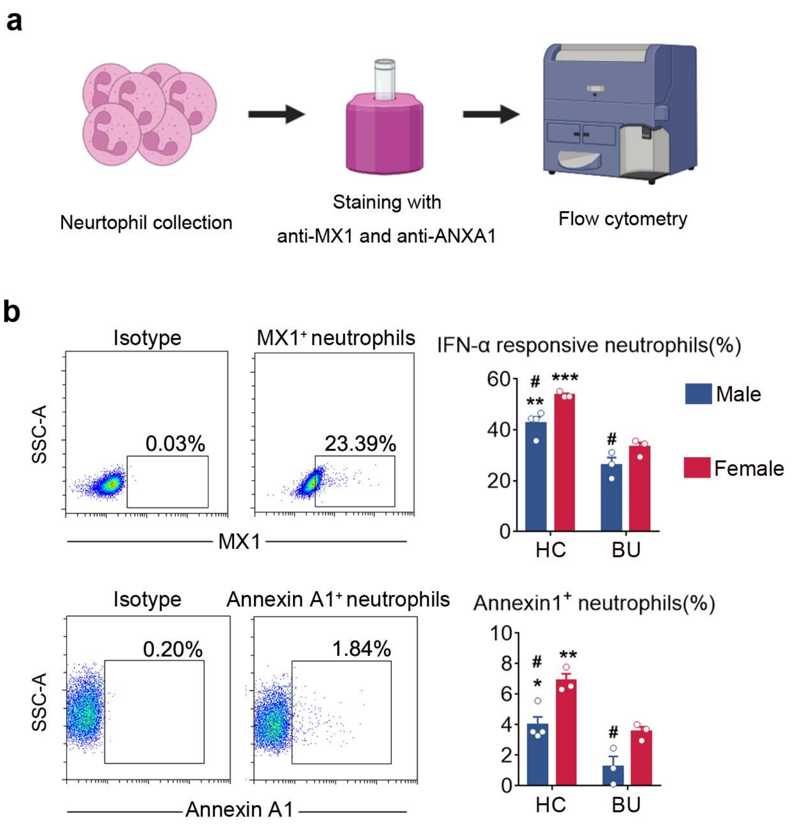

Fig S5. Flow cytometric analysis of unconventional neutrophil subsets.

a The experimental scheme of quantification of Annexin A1 and MX1 in human neutrophils using flow cytometry. **b** Sex-specific differences in the frequencies of $MX1^+$ neutrophils and Annexin $A1^+$ neutrophils between active BU patients (3 females and 3 males) and HCs (3 females and 4 males). In all instances, n refers to the number of each group. Mean and s.e.m. shown. Data were analyzed by the one-way ANOVA (Tukey's multiple comparison test). #females vs males; *patients vs HCs. *$P < 0.05$; **$P < 0.01$; ***$P < 0.001$; $^{\#}P < 0.05$.

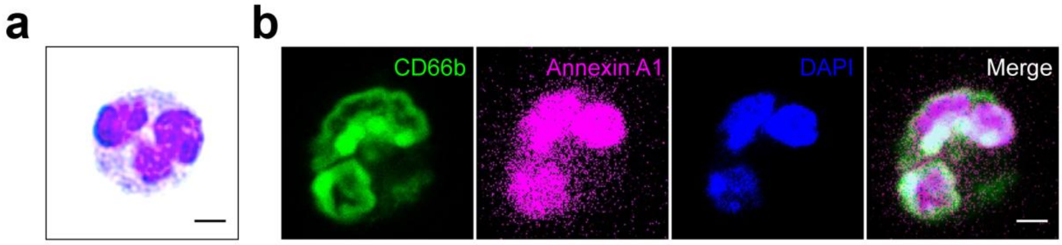

Fig S6. Wright-Giemsa and immunofluorescence staining of sorted human Annexin A1^{+} neutrophils.

a Representative images of Giemsa-stained cytospins of Annexin A1^{+} human neutrophils isolated by FACS. Scale bar: 2 μm, magnification: 63 ×. **b** Representative immunofluorescence staining images of human neutrophils sorted by FACS. The distribution of CD66b and Annexin A1 were represented by green and pink fluorescence, respectively. Nuclei was stained by DAPI and was represented by blue fluorescence. Scale bar: 2 μm, magnification: 63 ×.

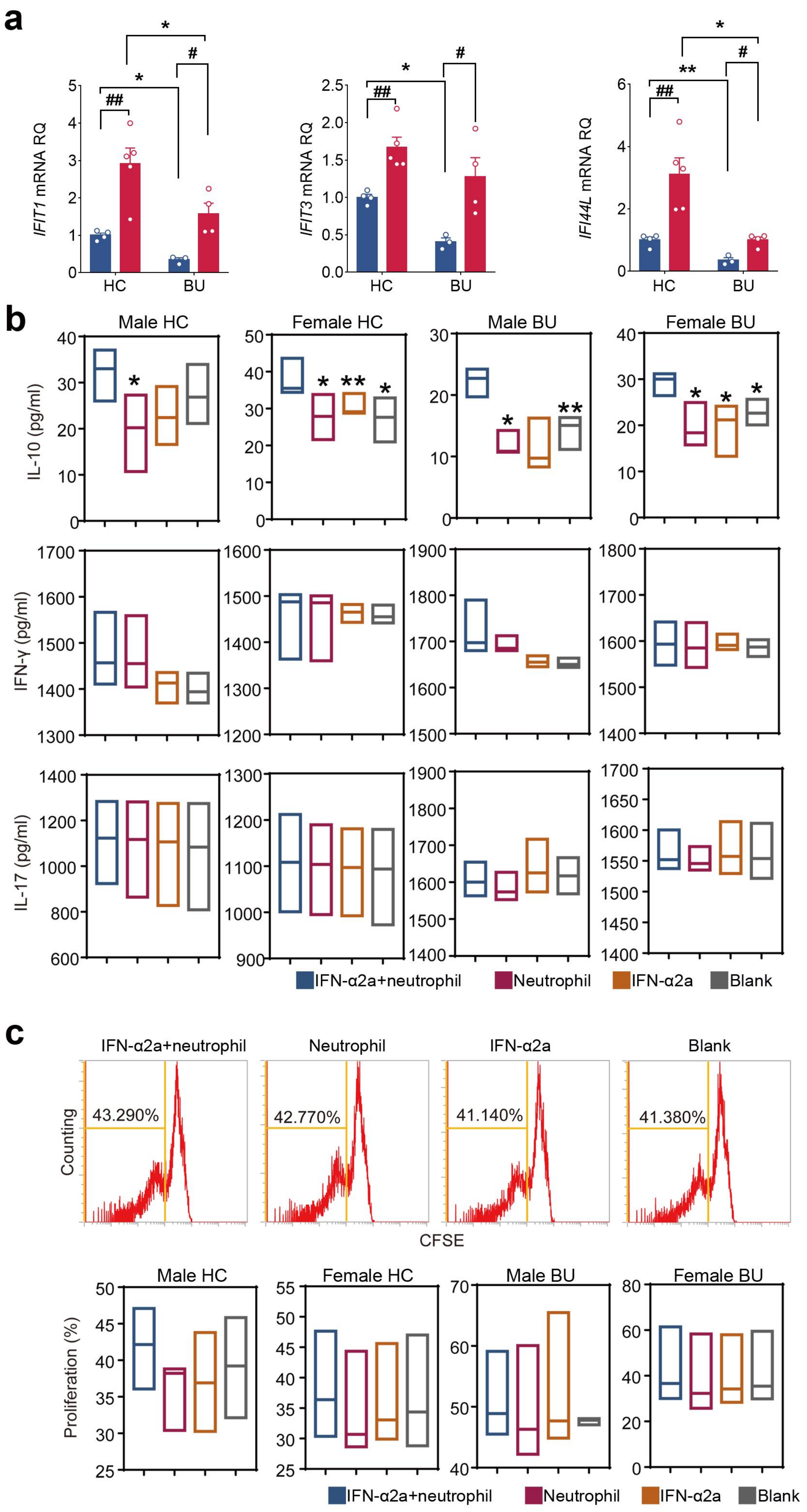
a
IFIT1 mRNA RQ
IFIT3 mRNA RQ
IFI44L mRNA RQ
HC
BU
##
#
*
**
b
Male HC
Female HC
Male BU
Female BU
IL-10 (pg/ml)
IFN-γ (pg/ml)
IL-17 (pg/ml)
IFN-α2a+neutrophil
Neutrophil
IFN-α2a
Blank
c
IFN-α2a+neutrophil
Neutrophil
IFN-α2a
Blank
Counting
43.290%
42.770%
41.140%
41.380%
CFSE
Male HC
Female HC
Male BU
Female BU
Proliferation (%)
IFN-α2a+neutrophil
Neutrophil
IFN-α2a
Blank

Fig S7. Co-culturing IFN-α2a pre-treated neutrophils with T cells.

a Sex-specific differences in the response to IFN-α2a of neutrophils from active BU patients (active BU patients, female: n = 4, male: n = 3; healthy individuals, female: n = 5, male: n = 4). Data are shown as mean ± SEM. *BU vs HC: *$P < 0.05$; **$P < 0.01$. #females vs males; #$P < 0.05$; ##$P < 0.01$. **b** The levels of IL-10, IFN-γ and IL-17 secreted by T cells after coculturing IFN-α2a pre-treated neutrophils with T cells (active BU patients, female: $n = 4$, male: $n = 3$; healthy individuals, female: $n = 5$, male: $n = 4$). **c** The frequency of proliferating T cells after coculturing IFN-α2a pre-treated neutrophils with T cells (active BU patients: female $n = 4$, male $n = 3$; healthy individuals: female $n = 5$, male $n = 4$). Data are shown as mean ± SEM. *$P < 0.05$; **$P < 0.01$. P values were calculated by the Wilcoxon matched pairs test.

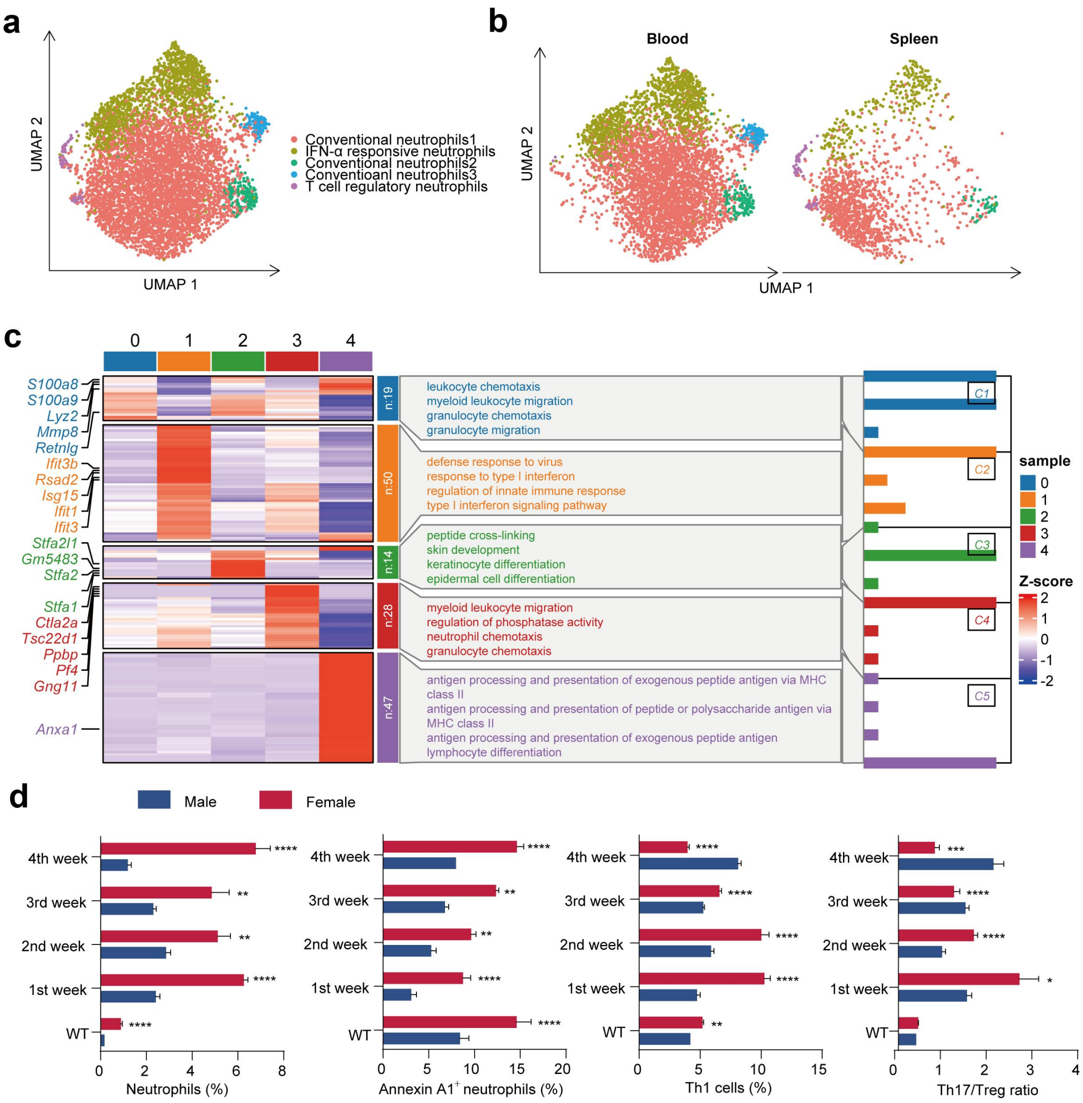

Fig S8. ScRNA-seq analysis and flow cytometric analysis of neutrophils in mice.

a UMAP visualization of neutrophils from WT mice. **b** UMAP visualization of splenic and blood neutrophils from WT mice. **c** Left panel: Heatmap showing expression of selected cluster marker genes in different neutrophil subsets; Right panel: GO biological process terms enriched in cluster marker genes. **d** Frequencies of splenic neutrophils and Annexin A1^{+} neutrophils in male and female EAU mice at 1, 2, 3, and 4 weeks after immunization (female EAU mice: *n* = 7 per group; male EAU mice: *n* = 8 per group). Vehical, mice without immunization (female mice *n* = 7, male mice *n* = 8). Data are shown as mean ± SEM. $*P < 0.05$; $**P < 0.01$; $***P < 0.001$; $****P < 0.0001$. *P* values were calculated by the Mann-Whitney U test.

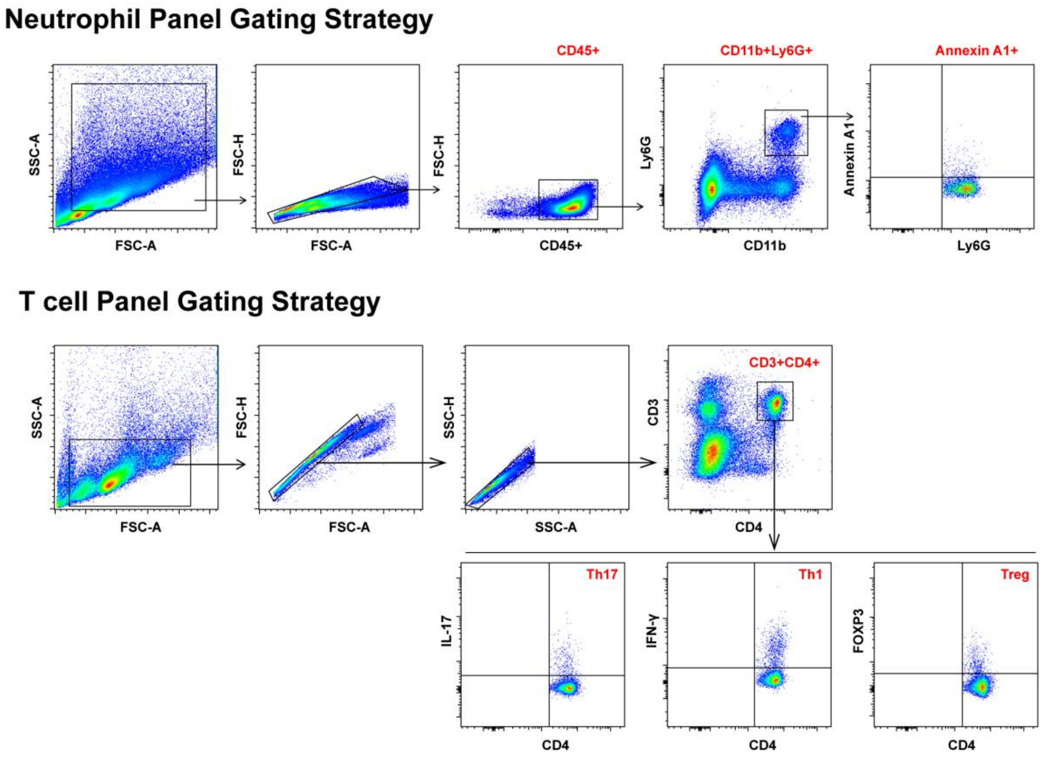

Fig S9. Gating strategy of neutrophils, Th1/Th17 cells, and Treg cells in mice.

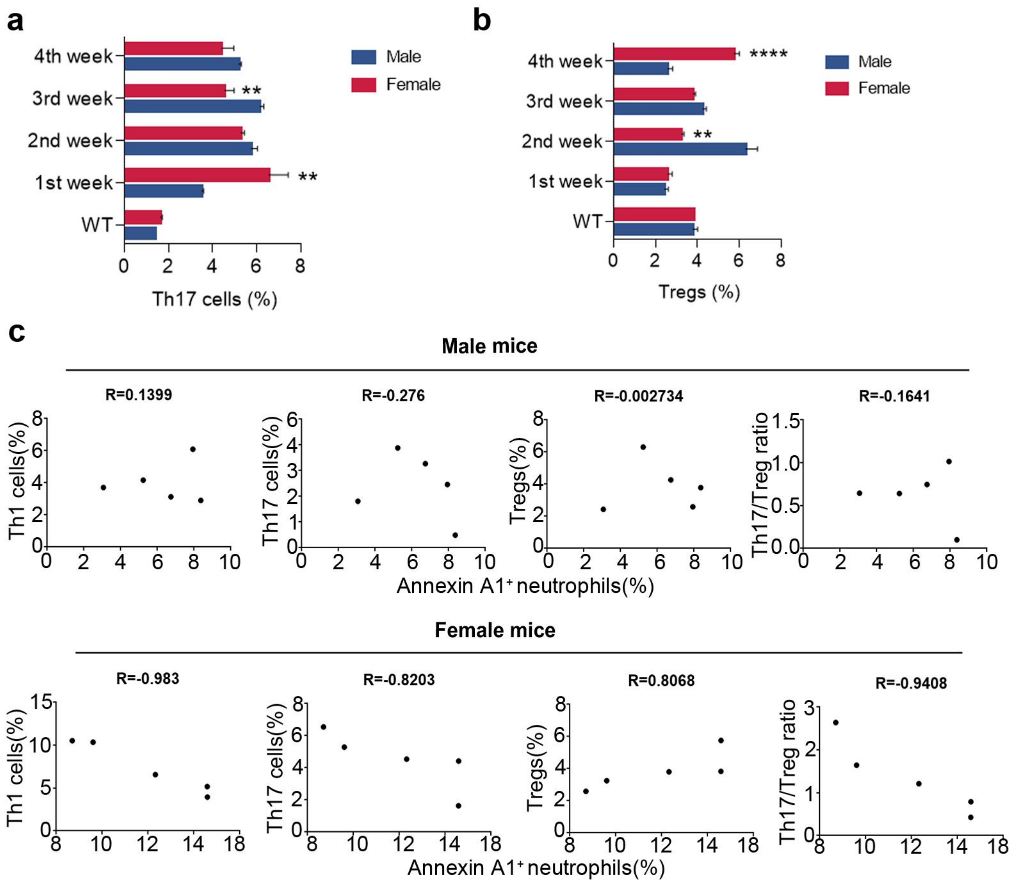

Fig S10. Sex-specific differences in the splenic T cell balance perturbation in EAU.

a and b Frequencies of splenic Tregs and Th17 cells in male and female EAU at 1, 2, 3, and 4 weeks after immunization (female EAU mice: n = 7 per group; male EAU mice: n = 8 per group). Vehical mice (female mice: n = 7, male mice: n = 8). **c** Upper panel: Correlation analysis on male EAU mice (n = 8) between Annexin $A1^+$ neutrophils and Th1/Th17 cells, Tregs, and Th17/Treg ratio, respectively; Lower panel: Correlation analysis on female EAU mice: (n = 7) between Annexin $A1^+$ neutrophils and Th1/Th17 cells, Tregs, and Th17/Treg ratio, respectively. Data are shown as mean ± SEM. $^{**}P < 0.01$; $^{****}P < 0.0001$. P values were calculated by the Mann-Whitney U test.

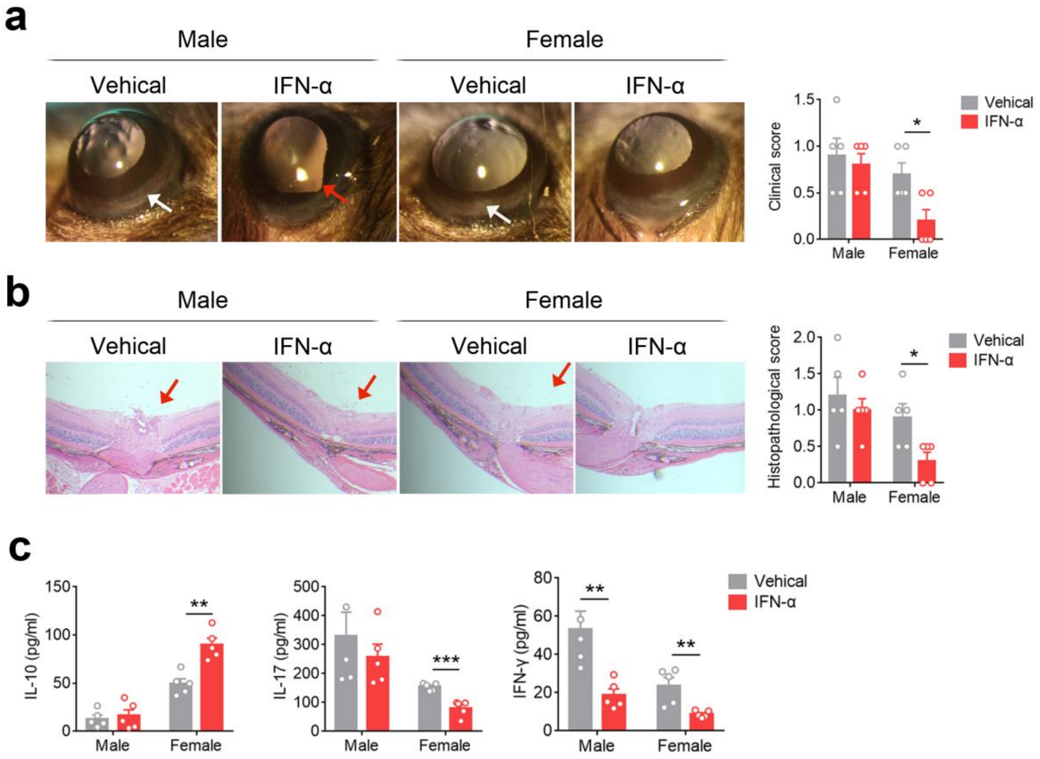

Fig S11. The effects of IFN-α2a treatment on the severity of EAU.

a The effects of IFN-α2a treatment on the clinical scores of male and female EAU at 4 weeks after immunization. $n = 5$ per group. White arrow, conjunctival and ciliary hyperemia. Red arrow, synechia. **b** The effects of IFN-α2a treatment on the histopathological scores of male and female EAU at 4 weeks after immunization. $n = 5$ per group. Red arrow, inflammatory cells. **c** The effects of IFN-α2a treatment on the production of IL-10, IL-17 and IFN-γ of male and female EAU at 4 weeks after immunization. Data are shown as mean ± SEM. $*P < 0.05$. P values were calculated by the Mann-Whitney U test.

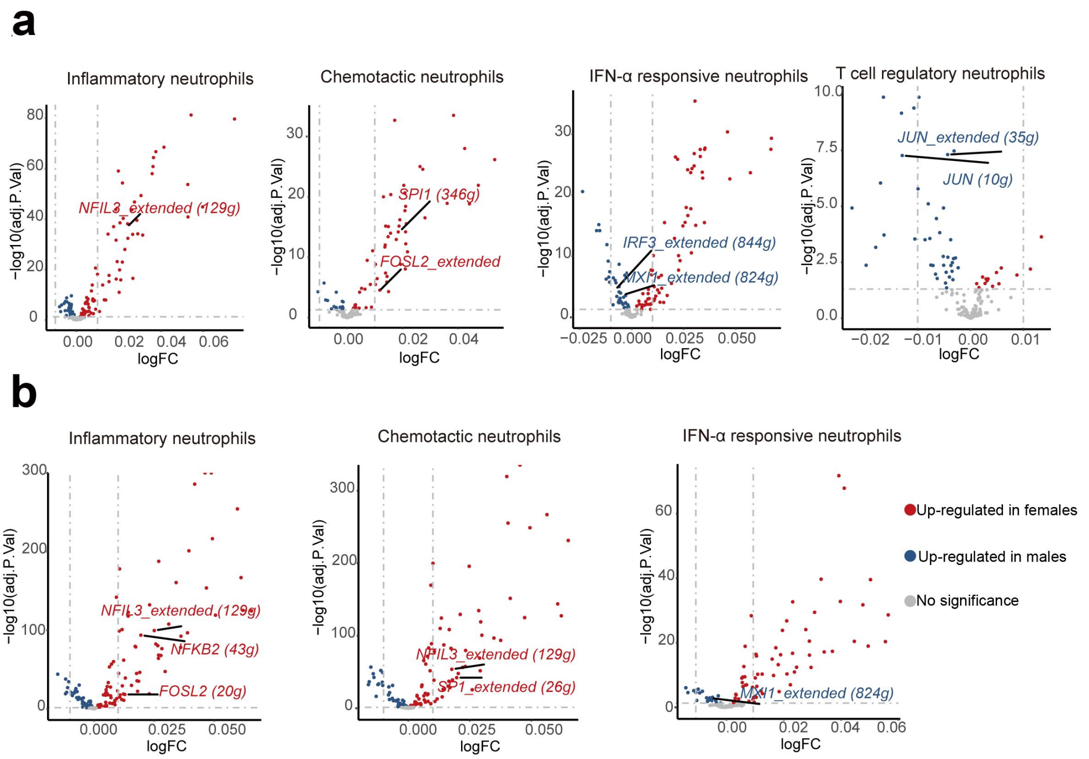

Fig S12. Sex differences in the TF expression within clusters.

a Sex differences in the TF expression within clusters from healthy individuals. **b** Sex differences in the TF expression within clusters from BU patients.
